# Unravelling specific diet and gut microbial contributions to inflammatory bowel disease

**DOI:** 10.21203/rs.3.rs-2518251/v1

**Published:** 2023-03-13

**Authors:** Gabriel Vasconcelos Pereira, Marie Boudaud, Mathis Wolter, Celeste Alexander, Alessandro De Sciscio, Erica. T. Grant, Bruno Caetano Trindade, Nicholas A. Pudlo, Shaleni Singh, Austin Campbell, Mengrou Shan, Li Zhang, Stéphanie Willieme, Kwi Kim, Trisha Denike-Duval, André Bleich, Thomas M. Schmidt, Lucy Kennedy, Costas A. Lyssiotis, Grace Y. Chen, Kathryn A. Eaton, Mahesh S. Desai, Eric C. Martens

**Affiliations:** 1 Department of Microbiology and Immunology, University of Michigan Medical School, Ann Arbor, Michigan, USA; 2 Department of Infection and Immunity, Luxembourg Institute of Health, 4354 Esch-sur-Alzette, Luxembourg; 3 Faculty of Science, Technology and Medicine, University of Luxembourg, 4365 Esch-sur-Alzette, Luxembourg; 4 Dept. of Internal Medicine, University of Michigan, Ann Arbor, Michigan, USA; 5 Unit for Laboratory Animal Medicine, University of Michigan, Ann Arbor, Michigan, USA; 6 Dept. of Ecology and Evolutionary Biology, University of Michigan, Ann Arbor, Michigan, USA; 7 Dept. of Molecular & Integrative Physiology, University of Michigan, Ann Arbor, Michigan, USA; 8 Institute for Laboratory Animal Science, Hanover Medical School, Hanover, Germany; 9 Odense Research Center for Anaphylaxis, Department of Dermatology and Allergy Center, Odense University Hospital, University of Southern Denmark, 5000 Odense, Denmark

## Abstract

Inflammatory bowel disease (IBD) is a chronic condition characterized by periods of spontaneous intestinal inflammation and is increasing in industrialized populations. Combined with host genetic predisposition, diet and gut bacteria are thought to be prominent features contributing to IBD, but little is known about the precise mechanisms involved. Here, we show that low dietary fiber promotes bacterial erosion of protective colonic mucus, leading to lethal colitis in mice lacking the IBD-associated cytokine, interleukin-10. Diet-induced inflammation is driven by mucin-degrading bacteria-mediated Th1 immune responses and is preceded by expansion of natural killer T cells and reduced immunoglobulin A coating of some bacteria. Surprisingly, an exclusive enteral nutrition diet, also lacking dietary fiber, reduced disease by increasing bacterial production of isobutyrate, which is dependent on the presence of a specific bacterial species, *Eubacterium rectale*. Our results illuminate a mechanistic framework using gnotobiotic mice to unravel the complex web of diet, host and microbial factors that influence IBD.

Inflammatory bowel disease (IBD) is characterized by periods of spontaneous inflammation in the gastrointestinal tract and occurs in people with underlying genetic variations that cause inappropriate immunological responses to intestinal antigens, especially ordinarily non-harmful symbiotic gut microbes [1]. Despite identification of hundreds of IBD-associated genetic polymorphisms [2], the precise mechanisms through which IBD develops have not yet been determined. Even when predisposing genetics exist, IBD does not always occur, suggesting that additional important triggers beyond host genetics and gut microbes are required [3].

The incidence of IBD is increasing in some industrializing countries [4] and in immigrant populations that move to already industrialized countries [5]. Dietary changes associated with industrialization (*e.g.*, decreased fiber intake, increased processed foods and emulsifiers) are emerging as potential “triggers” that enhance susceptibility to diseases like IBD [6-8], yet the underlying mechanisms of how dietary factors promote or suppress inflammation are still being determined. The physiology of the microbes inhabiting the gut is continuously influenced by diet, especially fiber polysaccharides that elude digestion in the upper gastrointestinal tract and arrive in the ileum and colon providing nutrients for microbes [9-11]. Several studies using murine models fed low fiber or “Westernized” diets have shown alterations in the microbiome that correlate with reduced integrity of the mucosal barrier, including increased activity of mucin-degrading bacteria and reduced mucus thickness [12, , 13] and increased mucus permeability [14].

Here, we investigated the specific contributions of dietary fiber and mucin-degrading gut bacteria to the development of spontaneous inflammation in mice lacking the human IBD-associated cytokine interleukin-10 (IL-10). In humans, loss of normal IL-10 production or either of its receptor subunits results in early onset IBD in infants and children [15]. Conventional *Il10^−/−^* mice develop spontaneous inflammation that is variable between mouse colonies and worsened by the presence of pathobionts like *Enterococcus faecalis* or *Helicobacter* spp. [16], while housing mice in specific-pathogen-free conditions [16] or deriving as germfree substantially reduces or eliminates inflammation [17]. It is therefore known that gut microbes are required for disease development in *Il10^−/−^* mice, however, the precise mechanisms potentiating disease progression in the presence of commensal bacteria that lack known or potential pathogenic qualities remain unknown.

Our data reveal that a combination of IL-10 loss, a gut microbiota containing mucin-degrading species and a low-fiber diet that increases bacterial mucus erosion are all required to elicit severe disease. At the same time, we fortuitously observed that the clinically validated dietary therapy exclusive enteral nutrition (EEN) partially inhibits colitis development, despite lacking dietary fiber, in part by promoting production of the bacterial metabolite isobutyrate. Our study represents an important step towards unraveling the positive and negative contributions of host genetics, gut microbial physiology and diet on a mechanistic level, and provides a potential path towards leveraging features of the IBD landscape that can be intentionally modified (diet, microbiome) to reduce the disease burden in people suffering from IBD.

## A combination of host genetic susceptibility, low dietary fiber and intestinal bacteria exacerbate colitis

**Extended Data Figure 1.**
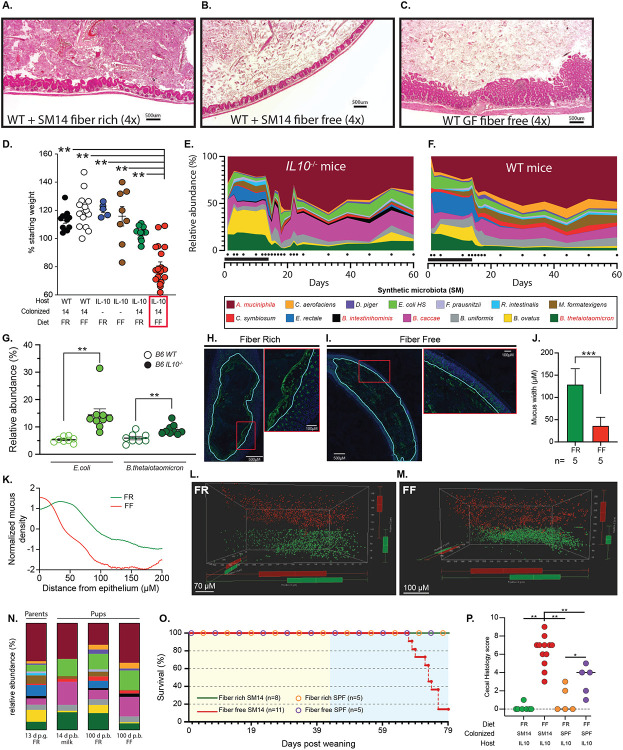
Low fiber and microbiome driven disease development in Il10^−/−^ mice. **A.-C.** Representative histology from wild-type (WT) SM14-colonized mice fed FR (A.), WT SM14-colonized mice fed FF (B.) or WT germfree mice fed FF (C.). All pictures are shown at 4x magnification, bars 500μM. **D.** Endpoint weights of the mice shown in Fig. 1A. Bold horizontal bar represents the mean and lighter error bars the S.E.M. (n=7–20, two-way ANOVA and post hoc test with Original FDR method of Benjamini and Hochberg) **E.–F.** Relative abundance streamplots of SM14 bacteria in Il10^−/−^ (E.) or wild-type (F.) mice shifted to the FF diet at 14d after colonization. Dots below the graph indicate the time points at which feces was sampled and the black bar on the x-axis represents the period of FR feeding. **G.** Comparison of cecal *E. coli* and *B. thetaiotaomicron* populations in Il10^−/−^ vs WT mice after 60d of colonization. Bold horizontal bar represents the mean and lighter error bars the S.E.M. (n=8-9, one-sample Student's t-test and Wilcoxon test) **H.–I.** Representative staining and imaging for Muc2 (green) in colonic sections from FR (H.) or FF (I.) and counter-stained with DAPI (blue). These images were used for automated mucus measurement using BacSpace software [13] as described in methods. **J.** Automated mucus thickness measurements generated from colonic section images like those shown in H.–I. (n=5, one-sample Student's t-test and Wilcoxon test) Five mice were measured per treatment, with a total of 1–2 fecal mass sections imaged per mouse. **K.** Averaged Muc2 staining intensity measurements for the mice represented in H.-J. as a function of distance from the epithelium. **L.–M.** Representative visualization of 1 um-sized beads (in red) layered on the distal colon epithelium (in green) after feeding FR (L.) or FF (M.). The space between the beads and epithelial cells represents the penetrability of the mucus layer. **N.** Representative SM14 composition in adult mice fed FR at 13d post gavage (p.g.) compared to still suckling pups at 14d post birth (p.b.) and 100d old SM14 colonized pups that were weaned onto either FR or FF diets. **O.** Survival plot of SM14 or SPF colonized pups weaned onto FR or FF diets and maintained for a total of 100d, the typical experimental duration. P. Cecal histology scores for SM14 or SPF colonized pups weaned onto FR or FF diets and maintained for a total of 100d. **P.** Cecal histology scores for SM14 or SPF colonized pups weaned onto FR or FF diets. Bold horizontal bar represents the mean and lighter error bars the S.E.M. (n=5–12, two-way ANOVA and post hoc test with Original FDR method of Benjamini and Hochberg).

**Figure 1.**
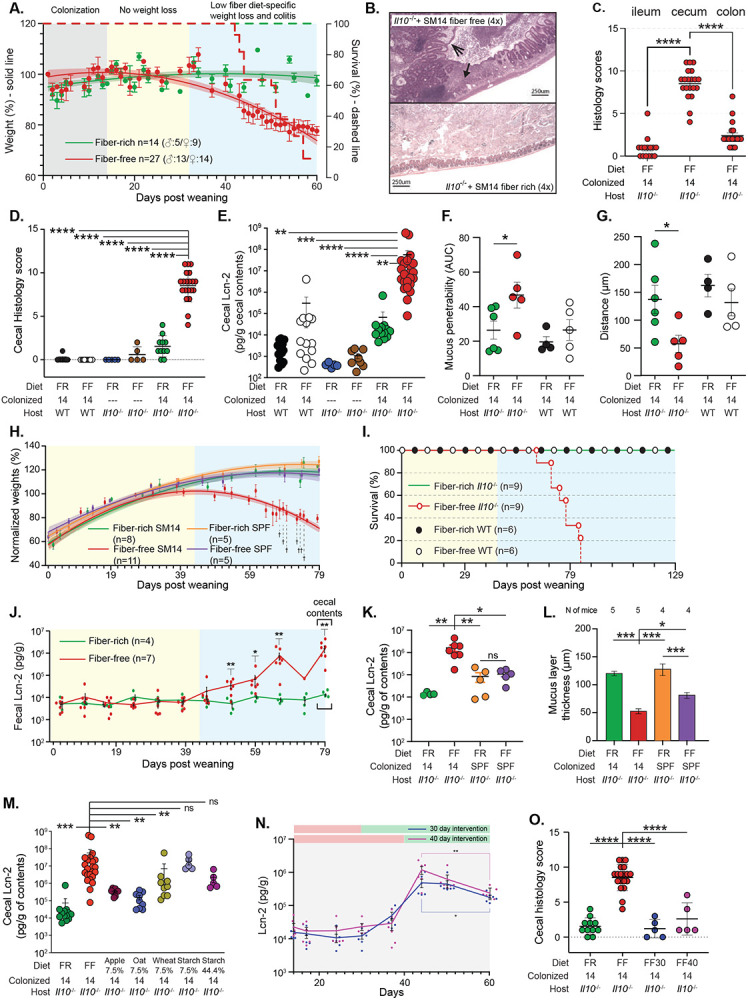
Low fiber and microbiome driven disease development in Il10^−/−^ mice. **A.** Weight trajectories of adult C57BL/6J Il10^−/−^ mice colonized with the human synthetic microbiota containing 14 species (SM14) and maintained on a fiber rich (FR) diet or switched to a fiber free (FF) diet after 14 d of colonization. Curves represent polynomial (quadratic) equations fit to all of the weights gathered at various days for the two treatments. Weights were measured more frequently after day 40 due to the declining health of the FF group and animals that were euthanized were excluded from the curve at later points. Two FF-fed animals from an early experiment were found dead on arrival and could not be included in subsequent analyses. All others were euthanized if they reached less than 80% starting weight and counted as lethality. The right axis shows survival in each group over time. The number of mice in each group, along with sexes are indicated in the figure legend. **B.** Representative cecal histology of FF (top) and FR (bottom) fed colonized IL10^−/−^ mice. In the top panel, block arrow points to a particularly large ulcer and the line arrow to an area of edema. Scale bars, 250 μm. **C.** Quantitative, blinded histological scoring of ileal, cecal, and colonic tissue taken from colonized Il10^−/−^ mice fed the FF diet. Bold horizontal bar represents the mean and lighter error bars the S.E.M. (n=15–20, one-way ANOVA and post hoc test with Holm-Šídák's multiple comparison test) **D.** Quantitative, blinded histological scoring of cecal tissue taken from colonized Il10^−/−^ mice fed the FR and FF diets, along with additional treatments discussed in the text to manipulate individual diet (FR/FF), colonization (SM14 or germfree, latter indicated by dashed lines) and host genotype (wild-type, WT, or Il10^−/−^) variables. Here, and in subsequent panels, the red box highlights the condition with most severe inflammation. Bold horizontal bar represents the mean and lighter error bars the S.E.M. (n=5–20, two-way ANOVA and post hoc test with Original FDR method of Benjamini and Hochberg) **E.** Cecal lipocalin (Lcn2) measurements in the ceca of animal treatments shown in D. Bold horizontal bar represents the mean and lighter error bars the S.E.M. (n=5–23, two-way ANOVA and post hoc test with Original FDR method of Benjamini and Hochberg) **F.** Mucus penetrability by 1 um-sized beads in the distal colon of Il10^−/−^ and WT mice fed the FR and FF diets. **G.** Distance of 1 um-sized beads from the epithelium in the same mice as in F. **H.** Weight trajectories of Il10^−/−^ mice born to either SM14 or specific pathogen free (SPF) parents and weaned to FR or FF. By 79d post weaning (100d after birth), only the SM14, FF group displays weight loss. Curves represent polynomial (quadratic) equations fit to all of the weights gathered at various days for the two treatments. **I.** Survival curves for 4 separate groups of WT C57bl/6 or Il10^−/−^ mice colonized by parental transfer of SM14 bacteria at birth. All groups show 100% survival until 129d post weaning (150d total), except for FF fed mice which experience 100% lethality by 105d. **J.** Fecal Lcn2 measurements over time in SM14 colonized Il10^−/−^ pups weaned to FR and FF. FF mice experience progressively increasing levels that are reciprocal with the weight trajectory shown in H. Middle horizontal bars represent the mean and flanking error bars the S.E.M. **K.** Endpoint cecal Lcn2 levels in SM14 and SPF colonized mice. Bold horizontal bar represents the mean and lighter error bars the S.E.M. (n=4–7, two-way ANOVA and post hoc test with Original FDR method of Benjamini and Hochberg) **L.** Mucus thickness measurements in SM14 and SPF colonized mice. Bars represent the mean and error bars the S.E.M. Sample size is indicated below each treatment group. (n=4–5, two-way ANOVA and post hoc test with Original FDR method of Benjamini and Hochberg) **M.** Cecal Lcn2 measurements in mice fed versions of the FF diet with glucose replaced by dietary fiber from apple, wheat, oat or soluble starch. Concentrations of each addition are noted below the ingredient. Bold horizontal bar represents the mean and lighter error bars the S.E.M. (n=5–23, two-way ANOVA and post hoc test with Original FDR method of Benjamini and Hochberg) **N.** Fecal Lcn2 measurements over time in SM14 colonized adult mice, which were shifted to FF at 14d post colonization and then shifted back to FR at either 30d or 40d. For each time point and treatment the mean is shown along with individual points and error bars represent S.E.M. **O.** Endpoint histological evaluation of the cecal tissue from mice shown in N. compared to mice maintained on FR or FF. (n=5–20, two-way ANOVA and post hoc test with Original FDR method of Benjamini and Hochberg) The experiments for data in panels F. and G. were done at the University of Luxembourg animal facility. All other experiments were done at the University of Michigan animal facility. P values: * < 0.05; ** < 0.01; ** < 0.001; *** < 0.001; **** < 0.0001; ns = not significant.

We previously determined that colonization of wild-type germfree mice with a synthetic microbiota containing 14 species (SM14), which is composed of sequenced and metabolically characterized human gut bacteria, results in erosion of the colonic mucus layer and increased pathogen susceptibility when mice are fed a fiber-free (FF) diet [12]. These fiber-deprived mice do not develop spontaneous inflammation and notably mice colonized with the same SM14 and fed high fiber harbor fewer mucin-degrading bacteria and do not experience mucus erosion. To determine if diet- and microbiota-driven mucus erosion—and the correspondingly increased proximity of bacteria to host tissue—promotes disease in mice that are genetically susceptible to IBD, we introduced the SM14 synthetic microbiota into germfree *Il10^−/−^* mice. Of note, *Il10^−/−^* lines have been constructed in several murine strain backgrounds and we chose the C57BL/6J derived line (mouse facility: University of Michigan), which has been reported to be the most resistant to inflammation development [16]. We colonized adult 7-10 week old *Il10^−/−^* mice fed a standard mouse chow, which we call a fiber-rich (FR) diet, for 14 days and switched a subset of the mice to the FF diet after 14 days of colonization, monitoring body weight and microbiota changes for up to 60d (Fig. 1A). Colonized mice that remained on the FR diet maintained or gained weight and did not experience noticeable morbidity. In contrast, mice switched to the FF diet began losing weight 1–2 weeks after the diet switch and experienced 88.9% lethality by 60d (n=27; note that a humane endpoint of ≥20% loss of starting weight was used and animals that exceeded this were counted as lethalities). Histological examination of the intestines of FR and FF fed mice at the end of the experiment (either 60d or sooner if mice succumbed to weight loss) revealed neutrophil infiltration, inflammation and epithelial damage that was most severe in the ceca of SM14-colonized FF-fed mice (Fig. 1B-D). Similar inflammation did not develop in wild-type mice with the same treatments (Fig. 1D, Fig. Extended Data Fig. 1A-C). Cecal measurements of neutrophil-derived lipocalin 2 (Lcn2) provided additional data that inflammation was most severe in SM14-colonized *Il10^−/−^* mice fed the FF diet (Fig. 1E) and generally mirrored trends in histology. Experimental treatments in which the individual variables for diet (FR, FF), colonization (SM14 or germfree) and host genotype (wild-type or *Il10^−/−^*) were systematically manipulated supported the conclusion that severe inflammation and weight loss only develops in the context of three conditions: IL-10 deficiency, SM14 colonization and low-fiber diet (Figs. 1D, E, Extended Data Fig. 1D).

As expected, switching SM14-colonized mice to the FF diet rapidly induced a microbiota shift in favor of decreased fiber-degrading and increased mucin-degrading species in *Il10^−/−^* mice (Extended Data Fig. 1E). The same trend was observed in wild-type mice fed the FF diet, albeit with significantly increased levels of *Escherichia coli* and *Bacteroides thetaiotaomicron* in *Il10^−/−^* mice (Extended Data Fig. 1E-G). Both of these species have previously been shown to benefit from an inflamed environment [18, 19], in some cases by sharing iron-scavenging siderophores [20]. As expected from studies with wild-type mice fed low fiber [12, 13], feeding the FF diet to *Il10^−/−^* mice resulted in reduced mucus thickness (Extended Data Fig. 1H-K) that likely increases bacterial contact with the host. Moreover, using bacteria-sized florescent beads in an *ex situ* mucus penetrability assay [21] we determined the colonic mucus penetrability. Our results revealed significantly higher mucus penetrability and closer proximity of 1μm-sized beads to the host epithelium in FF-fed *Il10^−/−^* mice compared to their FR-fed counterparts, an effect that was not as pronounced in the wild-type mice with SM14 (Fig. 1F, G, Extended Data Fig. 1L, M). Plating livers and spleens from colonized mice that suffered FF-induced disease on media that support growth of SM14 species did not reveal systemic bacterial dissemination (not shown), suggesting that intestinal inflammation, resulting from a damaged mucus barrier and a potentially increased influx of microbial antigens to the local intestinal tissue, is the main cause of mortality.

Human disease associated with IL-10 dysfunction often presents as early or very early onset IBD in children and infants [15] and GF mice have underdeveloped immune and mucosal barriers [22] that could contribute to the disease phenotype observed in colonized adult mice. With these points in mind, we modified our model to allow for microbiota transfer in the neonatal period by colonizing germfree *Il10^−/−^* adult parents fed the FR diet which allows pups to be exposed to the maternal microbiota beginning at birth. All 14 of the SM species were transmitted, although two of the more fastidious Firmicutes (*Faecalibacterium prausnitzii* and *Roseburia intestinalis*) were not transmitted to every pup, a phenomenon that did not appear to influence disease development. As anticipated, in pre-weaned, milk-fed pups born to FR-fed dams, the SM14 took on a composition that was similar to that observed in adult FF fed mice (Extended Data Fig. 1N) likely due to the lack of fiber and the fact that milk oligosaccharides share common several common glycosidic linkages with mucin *O*-glycans [23]. While there were no signs of disease development during the pre-weaning period, pups weaned to the FF diet began losing weight after ~40 days post weaning (dpw) (Fig. 1H) and experienced 100% mortality by 84 dpw (Fig. 1I). In most experiments, we harvested both FR and FF groups at 79 dpw (100 day old), in which case mortality among the FF mice was ~81.8% (Extended Data Fig. 1O). In contrast, all mice weaned to the FR diet survived to 79 dpw (Extended Data Fig. 1O) and a separate group of FR-fed mice showed 100% survival when maintained for 129 dpw (150 days total) as did wild-type mice fed either diet (Fig. 1I). Weight loss in pups weaned to the FF diet corresponded with increasing fecal Lcn2 that occurred around the same time (~40 dpw) of declining weight, further suggesting that weight loss is associated with increasing intestinal inflammation (Fig. 1J). Interestingly, *Il10^−/−^* mice with a specific pathogen free (SPF) microbiota and fed the FF diet did not lose weight as severely as those colonized with SM14 (Fig. 1H). Compared to SM14-colonized mice fed the FF diet, the SPF mice showed lower inflammation, measured by Lcn2, at 79 dpw (Fig. 1K), and less histological damage (Extended Data Fig. 1P), revealing that a more complex, murine microbiota does not promote the same level of disease in this model, a point that is addressed in more detail below. Interestingly, SPF mice fed the FF diet *did* exhibit reduced mucus thickness (Fig. 1L), although not as severe as SM14-colonized mice fed the FF diet, implying that it contains mucin-degrading bacteria capable of eroding this layer in a diet-dependent fashion but other factors may offset inflammation development.

**Extended Data Figure 2.**
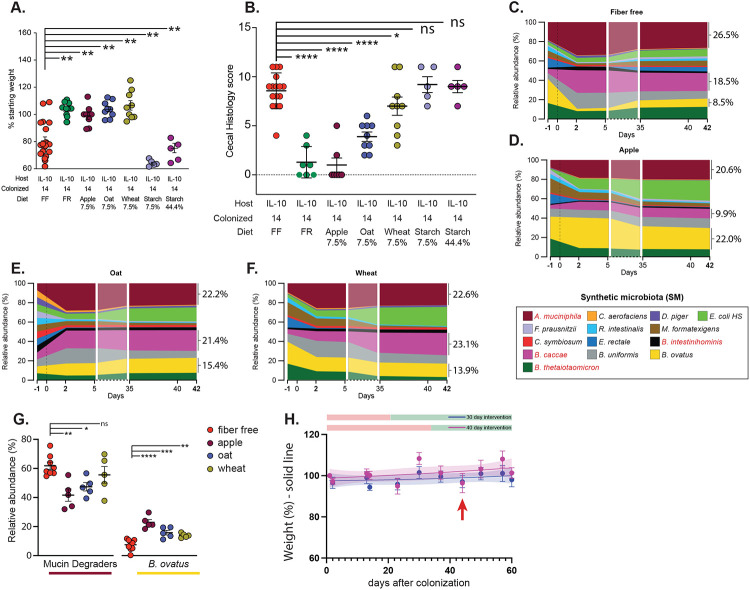
Fiber supplementation reduces inflammation severity. **A.** Endpoint weights of mice fed variations of the FF diet with glucose replaced by fiber from apple, oat or wheat, or soluble starch. Fiber or starch were added at the concentrations listed below each treatment and an equal amount of glucose was omitted. Bold horizontal bar represents the mean and lighter error bars the S.E.M. (n=5–20, two-way ANOVA and post hoc test with Original FDR method of Benjamini and Hochberg) **B.** Cecal histology scores of the treatment groups shown in A. (n=5–20, two-way ANOVA and post hoc test with Original FDR method of Benjamini and Hochberg) **C.–F.** Relative abundance streamplots of the SM14 bacteria in Il10^−/−^ mice fed fiber-enriched versions of the FF diet and containing either no added fiber (C.) or purified fiber from apple (D.), oat (E.) or wheat (F.). Relative abundance values for *A. muciniphila, B. caccae* and *B. ovatus* are shown next to each plot. **G.** Relative abundance comparison of all 4 mucin degraders compared to the fiber-degrader *B. ovatus* in adult mice colonized with SM14 and fed FF or the fiber supplemented diets. Note that the apple diet which had the lowest inflammation had the largest increase in *B. ovatus* and the largest decrease in mucin degrading bacteria. (n=5–8, two-way ANOVA and post hoc test with Original FDR method of Benjamini and Hochberg) H. Weight trajectories of the mice that were returned to the FR diet at either 30d or 40d. H. Weight trajectories of the mice shown in Fig. 1N, showing that no observable weight loss occurred prior to 30d or 40d fiber interventions.

The FR and FF diets differ substantially in several aspects of their macronutrient composition beyond fiber (Supplemental Table 1). To more directly test the contribution of fiber, we created modified versions of the FF diet in which 7.5% of the carbohydrate it contains (glucose) was removed and replaced with an equivalent amount of different, food grade purified fibers from oat, wheat or apple. A high sugar diet has been shown to promote inflammation, including in *Il10^−/−^* mice [24]. As a control for reducing the sugar concentration in our fiber supplemented diets, we created a diet that contained the same amount of highly digestible starch, effectively exchanging 7.5% of free glucose for a polymeric form of the same sugar that should still be available to the host via upper GI digestion. Our data reveal that the presence of 7.5% fiber from either of the three sources, but not digestible starch, reduces inflammation measured by Lcn2 (Fig. 1M) as well as weight loss and histopathology (Extended Data Fig. 2A, B). Even when all of the glucose (44%) was replaced with digestible starch, adult mice colonized with the SM14 developed disease (Fig. 1M, Extended Data Fig. 2A, B). *Bacteroides ovatus*, a proficient degrader of both the pectic and hemicellulosic polysaccharides expected to be present in the supplemental fibers used [25], was one of the major responders to the fibers used in these experiments, increasing between 2–3 fold in relative abundance in the community and this increased proliferation generally occurred at the expense of mucin-degrading species like *Akkermansia muciniphila* or *Bacteroides caccae* (Extended Data Fig. 2C-G).

To determine if restoring dietary fiber to mice that had already been fed the disease-promoting FF diet is capable of blocking inflammation, we returned colonized adult mice, which ordinarily experience ~89% lethality by 60d, to the FR diet at either 30d or 40d (16 or 26 days after being switched to FF). Both groups of mice that were returned to a high-fiber diet exhibited no lethal weight loss (Extended Data Fig. 2H) and Lcn2 levels and histology at 60d were significantly lower than mice maintained on the FF diet (Fig. 1N, O). Interestingly, fecal Lcn2 measurements over time showed that both groups of mice experienced a peak of inflammation after fiber had been restored, which then began declining, indicating that low fiber induces disruption to the host–microbe homeostasis in which inflammation is a lagging effect that can eventually be reset (Fig. 1N).

## The status of the mucus layer is a critical determinant of inflammation development

**Extended Data Figure 3.**
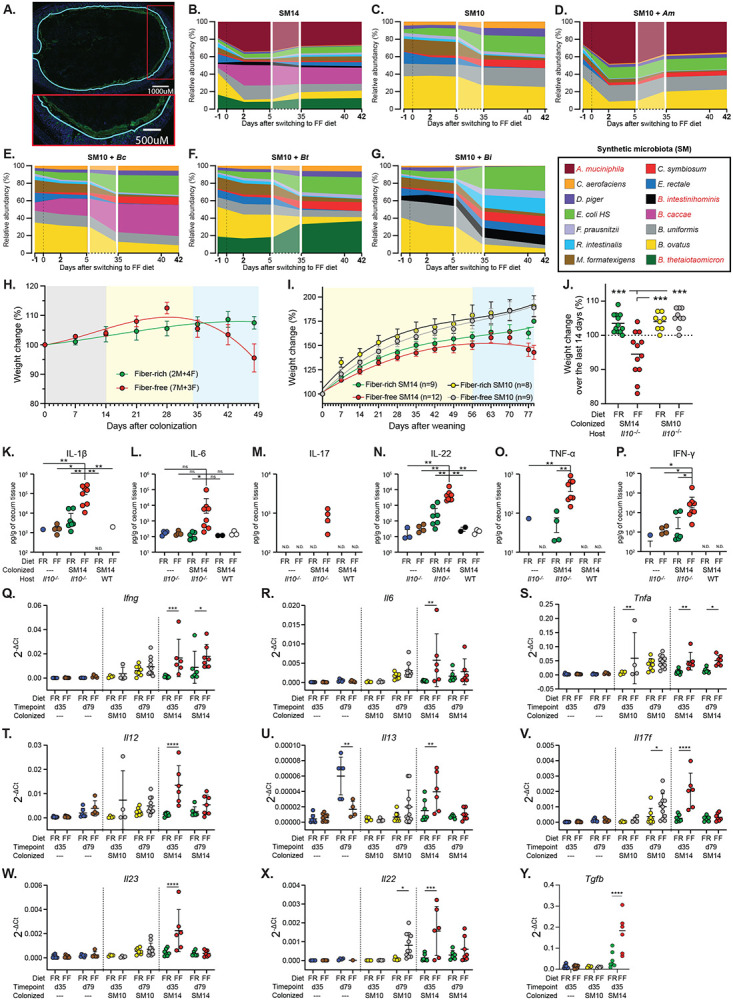
Elimination of mucin-degrading bacteria reduces inflammation development. **A.** Representative mucus staining for SM10 colonized mice used to measure thickness with Bacspace software. α-Muc2: green, DAPI: blue, prediction of epithelium/mucus interface: teal. **B.-G.** Relative abundance streamplots of the SM14 Il10^−/−^ mice with all bacteria present (B.), SM10 (C.), SM10 + *A. muciniphila* (D.), SM10 + *B. caccae* (E.), SM10 + *B. thetaiotaomicron* (F.), SM10 + *B. intestinihominis* (G.). **H.** Endpoint weights of mice with reduced presence of mucin-dergrading species. **I.** Weight trajectories of SM14 colonized adult Il10^−/−^ mice reproduced in a separate mouse facility (University of Luxembourg). **J.** Weight trajectories of SM14 and SM10 colonized Il10^−/−^ mice born to colonized dams and reproduced in a separate mouse facility (University of Luxembourg). **K.** Endpoint weight of individual animals shown in J. **K.-P.** Cytokine measurements by Luminex bead assay for wild-type and Il10^−/−^ mice with various colonization and diet treatment shown in main text Figure 1. **Q-Y**. Cytokine mRNA expression in the cecum of Il10−/− pups fed FF or FR diets. (n=4-11, two-way ANOVA and post hoc test with Original FDR method of Benjamini and Hochberg). Data are represented as mean ± SD. *p < 0.05; **p < 0.01; ***p < 0.001; ****p < 0.0001.

**Figure 2.**
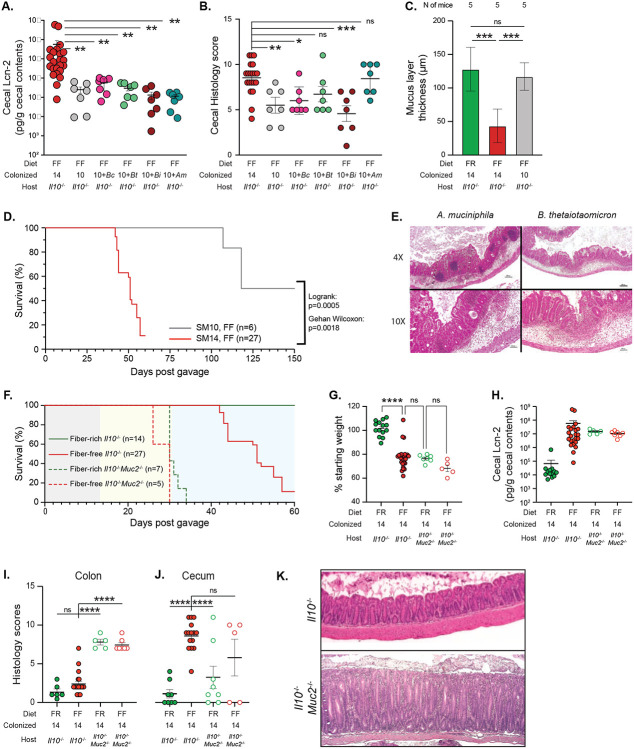
Mucus integrity is central to diet-induced inflammation development. **A.** Cecal lipocalin (Lcn2) measurements in Il10^−/−^ mice with full or reduced complexity synthetic microbiota as indicated in the “Colonized” line below each graph: Full 14 species SM (“14”), just the 10 species that do not degrade mucin *O*-glycans in vitro (“10”), or 10 non mucin-degrading species plus individual mucin degraders, *B. thetaiotaomicron (Bt), B. caccae (Bc), B. intestinhominis (Bi) or A. muciniphila (Am)*. Bold horizontal bar represents the mean and lighter error bars the S.E.M. (n=7–23, two-way ANOVA and post hoc test with Original FDR method of Benjamini and Hochberg) **B.** Cecal histology scores of the same treatment group shown in A. (n=5–20, two-way ANOVA and post hoc test with Original FDR method of Benjamini and Hochberg) **C.** Mucus thickness measurements of SM10 colonized mice fed the FF diet (gray) compared to SM14 colonized mice fed either diet. (n=5, two-way ANOVA and post hoc test with Original FDR method of Benjamini and Hochberg) **D.** Survival curves of Il10^−/−^ adult mice colonized with SM14 and SM10 to a maximum of 150d. **E.** Representative histology images of SM10, SM10+*Bt* and SM10+*Am*. **F.** Survival curves of Il10^−/−^ and DKO adult mice colonized with the SM14. **G.-J.** Individual endpoint weight loss (G.), Cecal Lcn2 (H.), colon histology (I.) and cecal histology (J.) for SM14-colonized Il10^−/−^Muc2^−/−^ double knockout (DkO) mice fed the FR or FF diets. In all three panels the mean is shown and error bars represent the S.E.M. (n=5-23, two-way ANOVA and post hoc test with Original FDR method of Benjamini and Hochberg) **K.** Comparison of colon histology in *Il10^−/−^* (top) and DKO (bottom) mice showing worse disease in DKO mice.

To more directly evaluate the disease-promoting role of mucin-degrading bacteria, we colonized adult *Il10^−/−^* mice with a more simple synthetic microbiota containing only the 10 species (“SM10”) that have previously been shown to be unable to grow on mucin oligosaccharides [12]. Mice colonized with a microbiota that lacked the four mucin degraders exhibited 100% survival on the FF diet until 60d post colonization (n=7), lower cecal Lcn2 at 60d (Fig. 2A) and reduced histological damage (Fig. 2B). Removal of the 4 known mucin-degrading bacteria also corresponded with decreased mucus erosion (Fig. 2C, Extended Data Fig. 3A), suggesting that the damage they inflict on mucus integrity may be causal to the increased disease activity when they are present. Consistent with this idea, SM10-colonized mice that were switched to the FF diet for a total of 150d after colonization (136d after switch to FF) exhibited significantly better survival (50%), revealing that the presence of mucin-degrading bacteria accelerates disease development but that the presence of non-mucin-degrading bacteria will eventually elicit disease in the context of the dysregulated *Il10^−/−^* immune system and fiber deprivation (Fig. 2D). Interestingly, adding back single mucin-degrading bacteria to the SM10 community did not yield the same level of inflammation observed with the full 14-member community, indicating that multiple species may act synergistically to promote more severe disease (Fig. 2A). Consistent with the idea that low dietary fiber intake promotes the general proliferation of mucin-degrading bacteria, the mice colonized with SM10 plus a single mucin degrader all exhibited expansion of that mucin-degrading bacterium after the diet switch compared to SM14-colonized mice (Extended Data Fig. 3B-G). This is in contrast to mice colonized with the full SM14, which typically show expansion of *Akkermansia muciniphila* and *B. caccae* in response to low fiber (Extended Data Fig. 3B). Despite a lack of weight loss (not shown) and lower Lcn2, the presence of either *B. thetaiotaomicron* or *A. muciniphila* as the sole mucin-degrader produced histological inflammation in the cecum that was statistically identical to the full SM14 (Fig. 2B,E).

As a separate test of the protective role for mucus in this colitis model, which could potentially be influenced by the presence of dietary fiber independently of bacteria, we bred *Il10^−/−^Muc2^−/−^* double knockout (DKO) germfree mice, colonized them with the SM14 and fed either the FR or FF diets. As expected, these mice lost weight quickly (100% needed to be sacrificed before 30d on FF and before 34d on FR; n=5 and 7, respectively) and showed severe inflammation and mortality regardless of diet (Fig. 2F-J). These results suggest that the high sugar content of the FF diet is not the main driver of the acute disease since the low sugar FR diet also allows inflammation when the mucus layer is genetically eliminated. Interestingly, inflammation in DKO mice developed throughout the lower intestine and was more severe in the colon than in the cecum (Fig. 2I-K). This is likely due to the mucus system being thinner and patchier in the cecum than in the colon [26]. Thus, when *Il10^−/−^* mice experience low fiber-induced mucus erosion, the secreted mucin barrier is likely to fail first in the cecum. In contrast, in DKO mice the uniform elimination of Muc2 appears to promote more widespread inflammation that worsens more quickly in the colon.

## Mucin-degrading bacteria influence inflammatory immune pathways in *Il10^−/−^* mice

Development of spontaneous intestinal inflammation is a complex process involving innate and adaptive immune responses, and the critical role of Th1/Th17 cells has been described in the *Il10^−/−^* colitis model in conventional/SPF settings (Keubler et al., 2015). Nevertheless, how specific microbial triggers influence the underlying immune pathways and how these immune responses develop temporally are less clear. We repeated the same experiments described above with a different line of *Il10^−/−^* mice in the same C57BL/6J background used above (mouse facility: University of Luxembourg) and observed similar weight loss in both adult and post-weaning FF-fed mice colonized with SM14 (Extended Data Fig. 3H-J), validating that these results are robust and repeatable in a second laboratory. Cytokine measurements in cecal tissues revealed expected increases in IBD-associated markers IL-1β, IL-6, IL-17, IL-22, IL-23, TNF-α and IFN-γ in fiber-deprived *Il10^−/−^* mice that were either colonized with SM14 as adults (Extended Data Fig. 3K-P) or colonized at birth from maternal exposure (Extended Data Fig. 3Q-Y). In addition, we observed that *Il10^−/−^* mice colonized maternally from birth with the SM10 lacking mucin degraders recapitulated the reduced disease phenotype observed in adults (Extended Data Fig. 3I, J). In these SM10-colonized mice, fiber deprivation only slightly induced cecal expression of IL-17, IL-22 and TNF-α, which were elevated in SM14-colonized mice (Extended Data Fig. 3Q-Y). Given the contrasting colitis phenotypes in FF-fed SM14- and SM10-colonized *Il10^−/−^* mice, our model provides opportunities to investigate the contributions of not only the microbial triggers but the regionality, timing and severity of underlying inflammatory immune pathways associated with increased microbial mucin foraging driven by fiber deficiency.

**Extended Data Figure 4.**
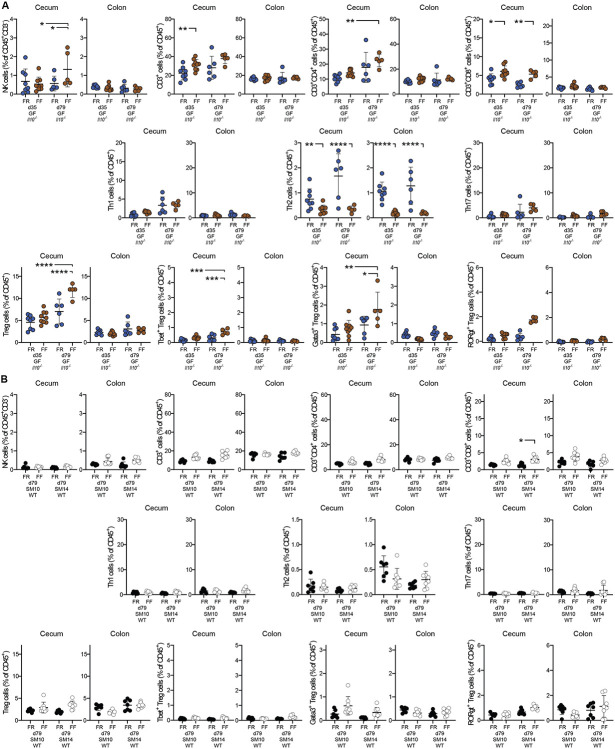
Fiber deprivation does not cause colitis in the absence of genetic predisposition and the microbiota. **A.–B.** Proportion of immune cell populations in the cecum and colon of: **A.** Germfree (GF) Il10^−/−^ mice and **B.** SM14- or SM10-colonized WT mice (n=5–9, two-way ANOVA and post hoc test with Original FDR method of Benjamini and Hochberg). Data are represented as mean ± SD and. For each cell population, data were analyzed along with data from Fig. 3 and S5. *p < 0.05; **p < 0.01; ***p < 0.001; ****p < 0.0001.

**Figure 3.**
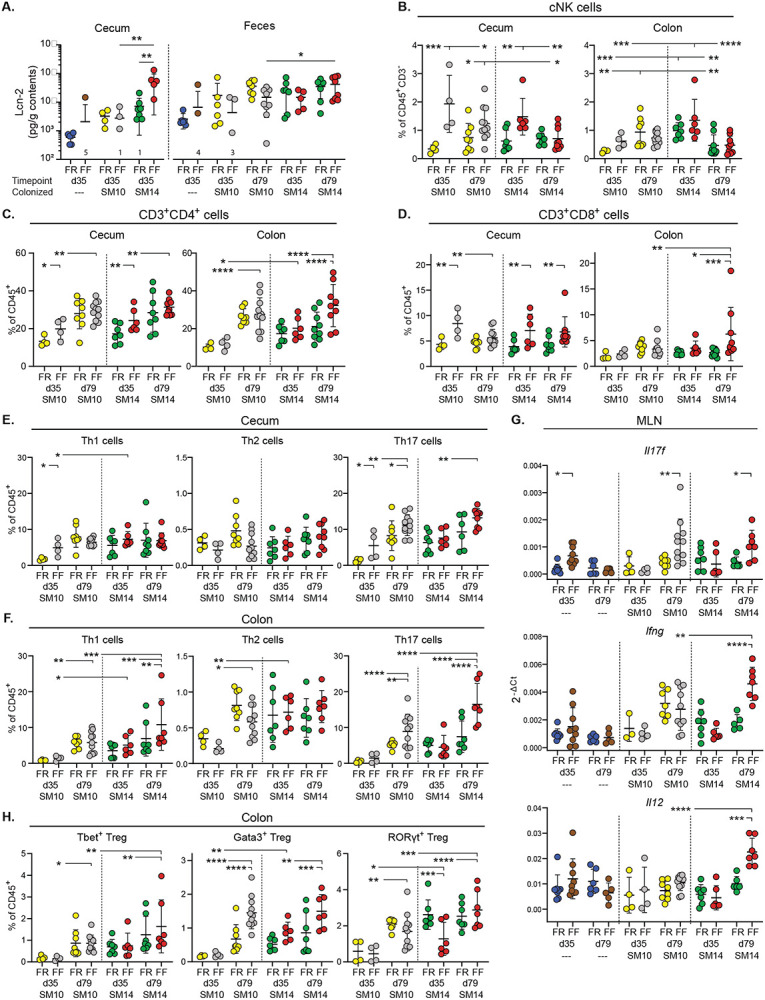
Mucolytic bacteria influence the inflammatory pathways of colitis. **A.** Lipocalin-2 levels in the cecal contents and feces of GF and colonized Il10^−/−^ mice (n=4–7, two-way ANOVA and post hoc test with Original FDR method of Benjamini and Hochberg). **B.** Proportion of NK cells among CD3-CD45+ cells in the cecum and colon lamina propria of SM10- and SM14-colonized Il10^−/−^ mice (n=4–11, two-way ANOVA and post hoc test with Original FDR method of Benjamini and Hochberg). **C.** Proportion of CD3+CD4+ cells among CD45+ cells in the cecum and colon of SM10- and SM14-colonized Il10^−/−^ mice (n=4–11, two-way ANOVA and post hoc test with Original FDR method of Benjamini and Hochberg). **D.** Proportion of CD3+CD8+ cells among CD45+ cells in the cecum and colon of SM10- and SM14-colonized Il10^−/−^ mice (n=4–11, two-way ANOVA and post hoc test with Original FDR method of Benjamini and Hochberg). **E.-F.** Proportion of helper T (Th) cell subsets (defined as CD3+ CD4+ Foxp3−) among CD45+ cells in the (E) cecum and (F) colon of SM10- and SM14-colonized Il10^−/−^ mice (n=4–11, two-way ANOVA and post hoc test with Original FDR method of Benjamini and Hochberg). **G.** Cytokine mRNA levels in the Mesenteric Lymph Nodes (MLN) of Il10^−/−^ mice (n=4–11, two-way ANOVA and post hoc test with Original FDR method of Benjamini and Hochberg). **H.** Proportion of regulatory T cell subsets among CD45+ cells in the colon of SM10- and SM14-colonized Il10^−/−^ mice (n=4–11, two-way ANOVA and post hoc test with Original FDR method of Benjamini and Hochberg). Data are represented as mean ± SD. *p < 0.05; **p < 0.01; ***p < 0.001; ****p < 0.0001.

In SM14-colonized mice that were weaned to the FF diet, inflammation could be detected in the cecum via increased Lcn2 as early as 35 dpw and this effect was absent in mice colonized from birth with SM10 (Fig. 3A). SM14 mice weaned onto the FF diet also exhibited low fecal Lcn2 at 35 dpw, consistent with data shown above and supporting the conclusion that disease develops earlier in the cecum in mice with intact mucus production (Fig. 3A). An expansion of natural killer (NK) cells was detectable in the cecum of FF-fed mice as soon as 35 dpw (Fig. 3B, **left panel**), and this diet-dependent expansion was similar in SM10- and SM14-colonized ceca. Interestingly, NK cells expanded in the colons of both FR and FF-fed SM14-colonized mice as soon as 35 dpw, but not in the colons of SM10-colonized mice (Fig. 3B, **right panel**). This observation further suggests that the presence of mucin-degrading bacteria is more important to elicit responses in the colon where the mucus system is thicker and mucus erosion is required to increase contact with bacterial antigens. Further supporting region-specific development of host responses, FF-fed germfree *Il10^−/−^* mice, but not WT germfree mice, showed increased NK cells in the cecum, but not in the colon, at 79 dpw compared to FR-fed controls, implying that long-term fiber deprivation can increase NK cell infiltration in the cecum independently of the microbiota (Extended Data Fig. 4A, B).

**Extended Data Figure 5.**
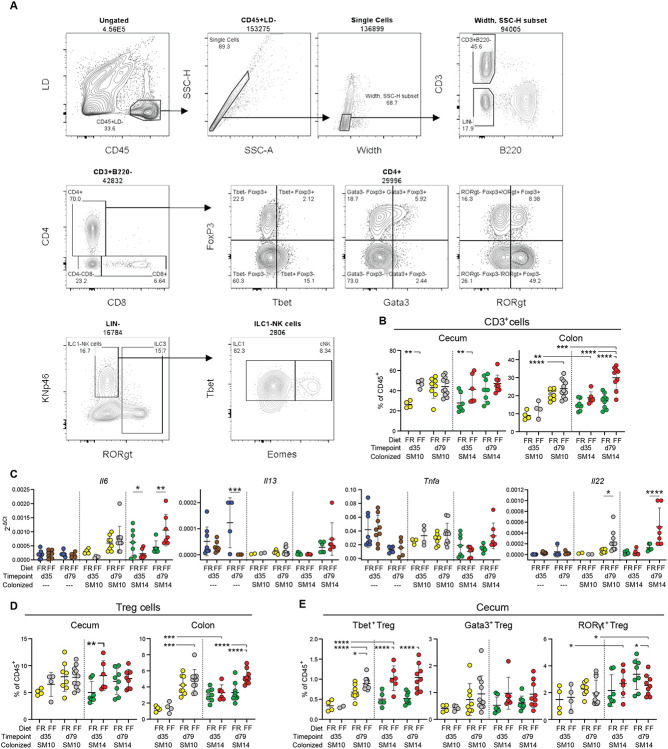
Mucin-degrading bacteria promote Th1 immune responses in a diet-dependent manner. **A.** Gating Strategy for the analysis of T cells and NK cells in the cecal and colonic lamina propria. **B.** Proportion of CD3+ cells among CD45+ cells in the cecum and colon of SM10- and SM14-colonized Il10^−/−^ mice (n=4–11, two-way ANOVA and post hoc test with Original FDR method of Benjamini and Hochberg). **C.** Cytokine mRNA levels in the Mesenteric Lymph Nodes (MLN) of Il10−/− mice (n=2–11, two-way ANOVA and post hoc test with Original FDR method of Benjamini and Hochberg). **D.–E**. Proportion of cecal and colonic regulatory T cells and (D.) and cecal regulatory T cell subsets (E.) among CD45+ cells in SM10- and SM14-colonized Il10^−/−^ mice (n=4–11, two-way ANOVA and post hoc test with original FDR method of Benjamini and Hochberg). Data are represented as mean ± SD. *p < 0.05; **p < 0.01; ***p < 0.001; ****p < 0.0001.

While NK cells were more abundant at 35 days and later decreased, T cell recruitment (especially CD4^+^ T cells) increased over time in both the cecum and colon of colonized *Il10^−/−^* mice (Fig. 3C and Extended Data Fig. 5A, B). In the ceca of colonized *Il10^−/−^* mice, fiber deprivation increased both CD4^+^ and CD8^+^ T cell populations by 35 dpw, with CD4^+^ T cells increasing further by 79 dpw independently of the diet (Fig. 3C, D). In the ceca of GF *Il10^−/−^* mice and SM14-colonized WT mice, CD8^+^ T cells and CD4^+^ T cells were also more abundant in FF-fed than in FR-fed mice, suggesting that fiber deprivation induces T cell recruitment through multiple paths and not all of these are regulated by the microbiota and IL-10 (Fig. 3D, E and Extended Data Fig. 4A,B).

Consistent with previous descriptions of *Il10^−/−^* colitis in conventional mice (Keubler etal., 2015), both SM10- and SM14-colonized mice generally exhibited infiltration of Th1 and Th17 cells over time. Especially for Th17 cells, this infiltration was higher in FF-fed mice in both the cecum (Fig. 3E) and the colon (Fig. 3F). While Th17 cells reached nearly equivalent levels at 79 dpw in SM10 and SM14-colonized ceca (Fig. 3E), overall levels were lower in the SM10-colonized colons compared to SM14. Furthermore, fiber deprivation increased Th1 levels in the SM14-colonized colons, but not in the SM10-colonized ones (Fig. 3F), consistent with the weight loss observed only in FF-fed SM14 colonized animals. In line with the readouts mentioned above, this observation supports the idea that the role of mucin-degrading bacteria is more important in the colon where erosion of mucus facilitates the induction of the anti-microbial Th1 responses. To provide functional support to these immune cell profiles, we compared levels of Th1- and Th17-type cytokine transcripts in ceca and mesenteric lymph nodes using qPCR for 35 and 79 dpw. Fiber deprivation led to increased expression of Th1 (IFN-γ, TNF-α, IL-6, IL-12) and Th17 cytokines (IL-17F, IL-22, IL-23 and TGF-β), as well as the mucin-inducing cytokine IL-13 in SM14-colonized *Il10^−/−^* mice (Extended Data Figs. 3Q-Y and 5C). These cytokine responses tended to develop more slowly in SM10-colonized ceca compared to those with SM14 (Extended Data Fig. 3Q-Y). In the colon-draining mesenteric lymph nodes (MLNs) where naïve T cells get activated and polarized, the Th17-related cytokines, IL-17F and IL-22, were increased by fiber deprivation in both SM10- and SM14-colonized mice, while the Th1-polarizing cytokines IFN-γ, IL-6 and IL-12 were only increased in SM14-colonized mice, consistent with a regional dependence on mucin-degrading bacteria to develop Th1 responses (Fig. 3G and Extended Data Fig. 5C).

Despite the IL-10 deficiency, Foxp3^+^ regulatory T cell (Treg) recruitment was also detectable and higher during FF feeding of SM14 *Il10^−/−^* mice (Fig. 3H, Extended Data Fig. 5D). This expansion being independent of IL-10 suggests a mechanism driven by other regulatory mediators such as TGF-β, whose transcript levels increased in SM14-colonized cecal tissues as soon as 35 dpw (Extended Data Fig. 3Y). Regulatory T cells expressing Tbet or RORγt have been proposed as counter-regulators of inflammatory Th1 and Th17 cells, respectively [27, 28]. Consistent with this, the abundance of Tbet^+^ Treg cells follow the same trends as inflammatory Th1 cells with higher levels in FF-fed, SM14-colonized mice (Fig. 3H and Extended Data Fig. 5E). By contrast, the highly suppressive RORγt^+^ Treg population was reduced at 35 dpw in the colon (Fig. 3H) and at 79 dpw in the cecum of FF-fed, SM14-colonized *Il10^−/−^* mice (Extended Data Fig. 5E), thus favoring the inflammatory responses. In addition, Gata3^+^ Tregs were increased in fiber-deprived germfree ceca (Extended Data Fig. 4B) and colonized colons (Fig. 3H). While their role in the specific regulation of type 2 inflammatory cells is still unclear, they may constitute a reservoir of Tregs required for tissue repair by 79 dpw in colonized colons [29, 30]. Finally, despite the general expansion of Treg, early (35 dpw) loss of the highly suppressive RORγt^+^ subset and the IL-10 deficiency are likely to allow the Th1/Th17 responses to flourish in FF-fed, SM14 mice. Together, these results reveal strong Th1/Th17 immune pathways induced by fiber deprivation, differentially regulated by microbial community members and host genetics, in a time- and intestinal site-specific manner.

## Alterations to IgA–microbiota interactions precede inflammation

**Extended Data Figure 6.**
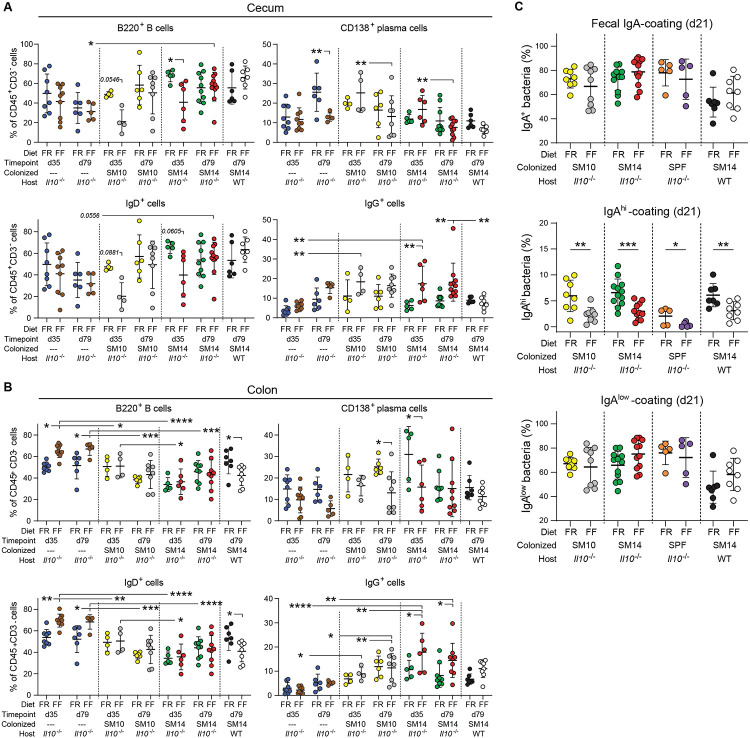
Fiber deprivation alters IgA–bacteria interactions. **A.–B.** Proportion of B cells (B220+), plasma cells (CD138+), IgD- and IgG-producing cells among CD3-CD45+ cells in the cecum (A.) and colon (B.) of GF, SM10- and SM14-colonized Il10^−/−^ and WT mice (n=4–11, two-way ANOVA and post hoc test with Original FDR method of Benjamini and Hochberg). **C.** Percentages of total (top), IgAhigh (middle) and IgAlow (bottom) -coated bacteria as shown in (C) in the feces of SM10-, SM14- or SPF-colonized Il10^−/−^ mice and SM14-colonized WT mice fed the FR or the FF diet for 21 days (n=5–8, two-way ANOVA and post hoc test with the two-stage linear step-up procedure of Benjamini, Krieger and Yekutieli). Data are represented as mean ± SD. *p < 0.05; **p < 0.01; ***p < 0.001; ****p < 0.0001.

**Figure 4.**
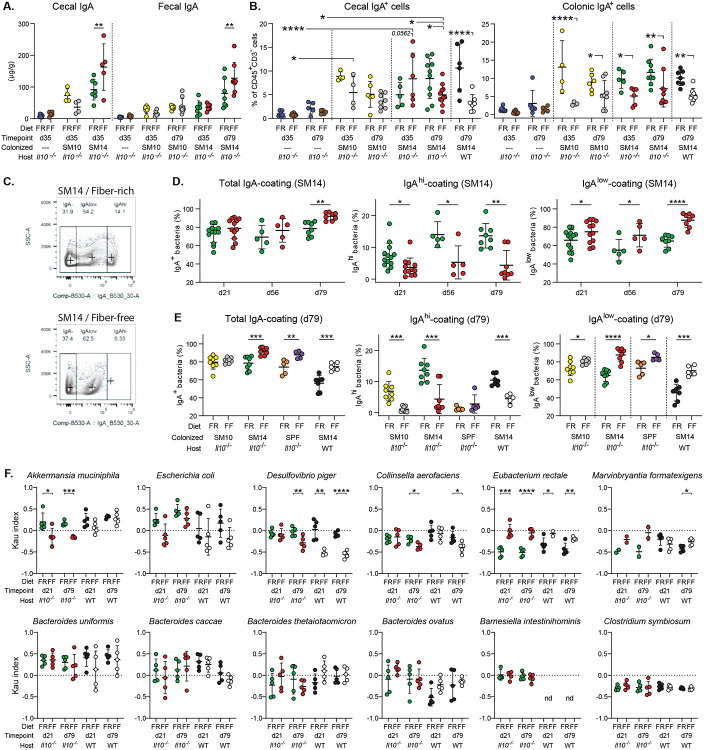
Fiber deprivation alters IgA-bacteria interactions in a microbial species-specific manner. **A.** Concentration of free IgA in the cecal content and feces of GF and colonized Il10^−/−^ mice (n=4–9, two-way ANOVA and post hoc test with the two-stage linear step-up procedure of Benjamini, Krieger and Yekutieli). **B.** Proportion of IgA-producing cells among CD3-CD45+ cells in the cecum (left) and colon (right) of GF SM10- and SM14-colonized Il10^−/−^ and WT mice (n=4–11, two-way ANOVA and post hoc test with the two-stage linear step-up procedure of Benjamini, Krieger and Yekutieli). **C.** IgA-coating profiles of fecal bacteria from SM14-colonized mice fed a FR (top) or a FF (bottom) diet for 56 days showing the gating strategy of population being low-coted (IgA^low^) and high-coated (IgA^high^). **D.-E.** Total IgA coating shown in panels D.–E. consists of the addition of IgA^high^ and IgA^low^ coating. (D) Percentages of total (left), IgA^high^ (middle) and IgA^low^ (right) -coated bacteria as shown in (C) in the feces of FR- and FF-fed, SM14-colonized mice at 21, 56 and 79 dpw (n=5–13, two-way ANOVA and post hoc test with the two-stage linear step-up procedure of Benjamini, Krieger and Yekutieli). (E) Percentages of total (left), IgA^high^ (middle) and IgA^low^ (right) -coated bacteria as shown in (C) in the feces of SM10-, SM14- or SPF-colonized Il10^−/−^ mice and SM14-colonized WT mice fed the FR or the FF diet for 79 days (n=5–8, two-way ANOVA and post hoc test with the two-stage linear step-up procedure of Benjamini, Krieger and Yekutieli). **F.** IgA-coating index (Kau index) of fecal bacteria from SM14-colonized Il10^−/−^ and WT mice (n=2–5, multiple unpaired t-test and post hoc test with the two-stage linear step-up procedure of Benjamini, Krieger and Yekutieli). Data are represented as mean ± SD. *p < 0.05; **p < 0.01; ***p < 0.001; ****p < 0.0001.

Since coating with immunoglobulin A (IgA) has been proposed to identify bacteria that are potentially more colitogenic [31], we next focused on how low fiber-induced inflammation development alters bacterial IgA coating in our *Il10^−/−^* model. The IgA response is a common anti-microbial response in the gut and is usually upregulated in colitis in human and mouse models [32]. Mirroring the trend observed for early inflammation (Fig. 3A), soluble IgA titers were increased in the cecum but not feces of FF-fed SM14-colonized *Il10^−/−^* mice at 35 dpw compared to FR (Fig. 4A). However, prolonged FF-feeding (79 dpw) resulted in depletion of IgA-producing plasma cells in both the cecum and colon (Fig. 4B). This loss was also observed in FF-fed WT mice that were colonized with SM14 (Fig. 4B), suggesting a diet-driven effect on IgA-producing cell loss rather than a depletion caused by the absence of IL-10. This is consistent with a published report showing reduced titers of serum IgA and less IgA^+^ B cells in the small intestine of wild-type mice fed a zero-fiber diet compared to a high-fiber diet [33]. In parallel with reduced IgA-producing cells after FF-feeding, the proportion of IgG-producing cells was increased at 35 and 79 dpw in both the cecum and colon of SM14-colonized *Il10^−/−^* mice (Extended Data Fig. 6A,B). Consistent with the high production of Th1 cytokines in the presence of mucolytic bacteria, FF-feeding increased the proportion of IgG-producing cells only in SM14- but not in SM10-colonized colons (Extended Data Fig. 6B) [34].

We next sought to determine how changes in IgA-producing cells are reflected in bacterial IgA coating. Intriguingly, analysis of IgA-coated bacteria revealed the presence of 2 differentially coated populations in FR-fed mice: a large population with low-coating and a smaller population with high coating and we observed that the highly coated population was nearly absent in SM14-colonized mice fed the FF diet (Fig. 4C). Consistent with previous studies [32], the amount of total IgA-coated bacteria (both populations combined) was higher in FF-fed mice than in FR-fed mice by 79 dpw (Fig. 4D, **left**). However, only the proportion of low-coated bacteria increased in FF-fed mice (Fig. 4D, **right**), while the highly-coated bacteria were diminished in fiber-deprived mice, as early as 21 days of feeding (Fig. 4D, **middle**). Interestingly, the FF diet-induced increase in total IgA-coating by 79 dpw was not observed in SM10-colonized mice, but was observed in SPF *Il10^−/−^* mice and SM14-colonized WT mice, supporting a mechanism driven by the microbiota rather than the IL-10 deficiency (Fig. 4E, **left**). However, highly coated bacteria were diminished in SM10-colonized mice as in SM14-colonized mice as soon as 21 dpw (Fig. 4E, **middle and** Extended Data Fig. 6C) and low-coated bacteria only slightly increased by 79 dpw (Fig 4E, **right**), suggesting a mechanism that is partly driven by the presence of mucolytic bacteria.

Given the altered pattern of IgA coating and the fact that IgA coating has been proposed as a marker of bacteria with more colitogenic potential [31], we examined the IgA coating of individual SM14 bacterial members. Consistent with the overall early loss of highly coated bacteria, changes in IgA-coating indexes in the SM14 community appeared as soon as 21 days of feeding (Fig. 4F). Among the SM14 members, *A. muciniphila, D. piger, E. coli* and *C. aerofaciens* showed reduced IgA coating index (ICI) values at one or both timepoints in FF-fed *Il10^−/−^* mice. In wild-type mice, this FF diet-induced reduction in IgA coating was less severe for *A. muciniphila*, but more pronounced for *D. piger* and *C. aerofaciens*, revealing that IL-10 deficiency affects the IgA-coating profile of commensals in a species-dependent manner. In contrast, *E. rectale* showed a low ICI in FR-fed mice and this increased with FF-feeding, a condition that corresponds to it being present at very low levels (and therefore not contributing much to overall coating measurements), likely due to its inability to compete for mucin-derived nutrients. The causes and effects of variations in IgA coating are still being determined. However, our results demonstrate that different combinations of microbial colonization, diet and host immune status can alter both the amount and affinity of intestinal IgA, as well as the bacteria that it targets. Along with the data shown above, our results suggest the possibility that fiber deprivation initiates early disruptions in IgA–microbiota interactions along with a loss of IgA-producing plasma cells allowing *A. muciniphila* and *E. coli* to expand in the absence of proper IgA coating and contribute to colitis.

## Different microbiota alter the outcome of diet-induced colitis in *Il10^−/−^* mice

**Extended Data Figure 7.**
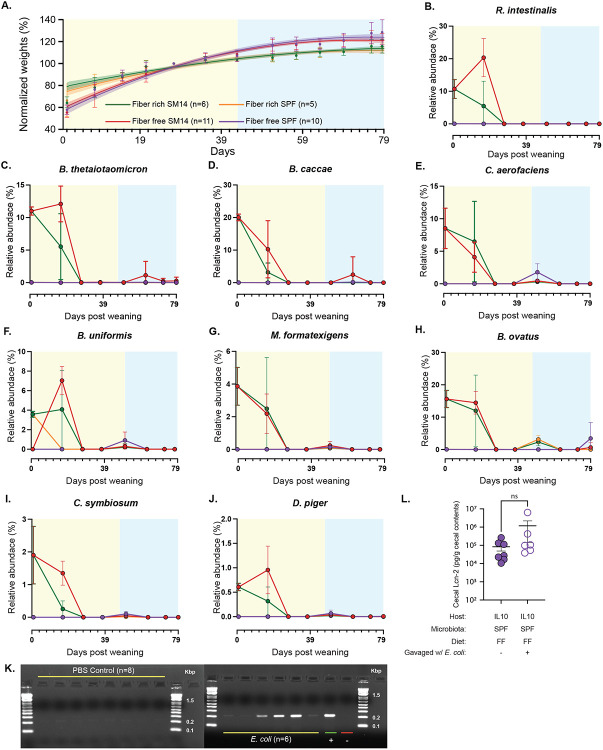
Effects of co-housing SM14 and SPF mice fed FR and FF diets. **A.** Weight trajectories of SM14 or SPF colonized mice that were co-housed at weaning (21d) with pups harboring the opposite colonization (SM14 vs. SPF), noting the weight loss typically observed in SM14/FF mice is eliminated. **B.-J.** 16S rRNA gene based measurements of the remaining SM14 bacteria in co-housed mice: *R. intestinalis* (B.), *B. thetaiotaomicron* (C.), *B. caccae* (D.), *C. aerofaciens* (E.), *B. uniformis* (F.), *M. formatexigens* (G.), *B. ovatus* (H.), *C. symbiosum* (I.) and *D. piger* (J.). **L.** Cecal lipocalin measurements in SPF mice that were gavaged weekly with *E. coli* HS and fed the FF diet. (n=6-7, one-sample Student's t-test and Wilcoxon test). **K.** PCR analysis using *E. coli* HS specific primers [12] for the presence of *E. coli* HS in mice that were mock gavaged weekly with PBS (left) or *E. coli* HS (right).

**Figure 5.**
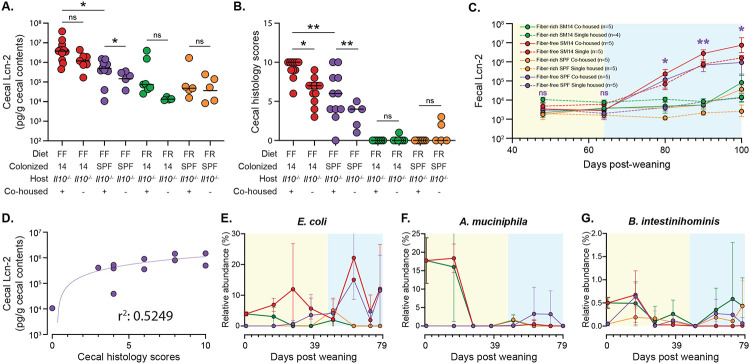
Co-housing SPF mice with SM14 colonized mice after weaning worsens disease. **A.** Cecal lipocalin (Lcn2) levels in co-housed or non-co-housed SPF and SM14 mice (co-housing status is indicated at the bottom of this and subsequent panels as appropriate). (n=5-11, one-sample Student's t-test and Wilcoxon test) **B.** Cecal histology of the same treatments shown in A. (n=5-11, one-sample Student's t-test and Wilcoxon test) **C.** Cecal Lcn2 over time in co-housed and non-co-housed SPF and SM14 mice showing inflammatory response starting between 60–80d. Note the difference in the group born SPF when co-housed or not and shown in solid or dashed purple lines. **D.** Correlation of cecal Lcn2 and cecum histology scores of co-housed SM14/SPF fed FF diet showing similarity between these two measurements in individuals. **E.–G.** Relative abundance of SM14 species invading SPF co-housed mice, *E. coli, A. muciniphila, B. intestinihominis*, respectively. Data are represented as mean ± SEM. *p < 0.05; **p < 0.01; ***p < 0.001; ****p < 0.0001.

Because we observed that SPF mice fed the FF diet did not develop inflammation with the same severity as those colonized with SM14 (Fig. 1H,K), despite having thinner mucus (Fig. 1L), we performed co-housing experiments in which pups born to SPF- and SM14-colonized mothers were mixed at weaning and exposed to each other’s microbiomes. Co-housing SM14-colonized *Il10^−/−^* pups with SPF mice prevented the weight loss phenotype observed in response to feeding the FF diet (Extended Data Fig. 7A). However, it did not reduce cecal inflammation as measured by both cecal Lcn2 (Fig. 5A) or histology (Fig. 5B), indicating that the weight loss and inflammation phenotypes can be uncoupled in the presence of different microbes perhaps due to the timing of barrier disruption or other changes in microbiota metabolism. Mice that were born to SPF mothers showed a more variable response to co-housing with SM14-colonized pups, with only some of these mice developing disease that was often similar in severity to FF-fed, SM14-colonized mice (Fig. 5A,B). Time course analysis of fecal Lcn2 revealed a larger difference between co-housed and non-co-housed SPF mice than cecal measurements and this marker increased in co-housed SPF mice after 65 days, a trend that was nearly identical to SM14 mice (Fig. 5C). Although inflammation was variable in co-housed SPF mice, there was a positive correlation in the two inflammatory measurements for this group (Fig. 5D).

The SPF pups are naturally colonized with a natively complex microbiota beginning at birth, whereas the SM14 pups receive a simpler microbiota of known composition. The fact that co-housed SPF pups develop worse inflammation compared to their littermates that are not co-housed suggested that some of the SM14 bacteria can invade the murine SPF microbiota and worsening disease, perhaps synergizing with native murine microbes that perform some of the same metabolic or immunostimulatory functions of other SM14 members. To determine which SM14 species invaded the SPF microbiota and when, we performed 16S rRNA gene sequencing on fecal samples from co-housed mice from both groups over the 100d time course. Our analysis revealed that only some of the SM14 bacteria are transferred to SPF mice, a finding that might be expected with human gut bacteria invading a more complex, mouse-adapted microbiota. However, at least 6 members of the SM14 could be detected in co-housed SPF mice, often transiently or at the very end of the time course when inflammation had begun to develop (Fig. 5E-G, Extended Data Fig. 7B-J). The most prominent of these invading bacteria was the human commensal *E. coli* strain HS, which appeared in co-housed SPF mice around 22 dpw, prior to the onset of inflammation, and gradually increased, eventually reaching levels >10% in most mice (Fig. 5E). Two of the mucin-degrading SM14 bacteria (*A. muciniphila* and *Ba. intestinihominis*) showed a similar trend, albeit reaching lower levels and with variability among individual mice (Fig. 5F,G). Four other SM14 bacteria (*C. aerofaciens, B. uniformis, B. thetaiotaomicron* and *B. caccae*) showed transient, small increases around the time that inflammation was increasing (~44 dpw) and these organisms decreased thereafter (Extended Data Fig. 7B-J). A test of whether or not weekly gavages of *E. coli* HS into SPF mice that were otherwise not exposed to additional SM14 bacteria did not support that conclusion that this species is the sole cause of increased inflammation (Extended Data Fig. 7K,L), suggesting that more complex microbial interactions are involved.

## Certain gut bacteria and metabolites associated with exclusive enteral nutrition prevent inflammation

**Extended Data Figure 8.**
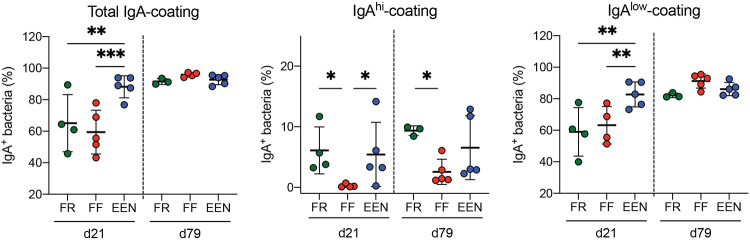
The EEN diet affects IgA coating differently than FR and FF feeding. Percentages of total (left), IgA^high^ (middle) and IgA^low^ (right) -coated bacteria as shown in Fig. 4C in the feces of SM14-colonized Il10^−/−^ mice fed the FR, FF or EEN diet for 21 or 79 days (n=3–5, two-way ANOVA and post hoc test with the two-stage linear step-up procedure of Benjamini, Krieger and Yekutieli). Data are represented as mean ± SD. *p < 0.05; **p < 0.01; ***p < 0.001.

**Figure 6.**
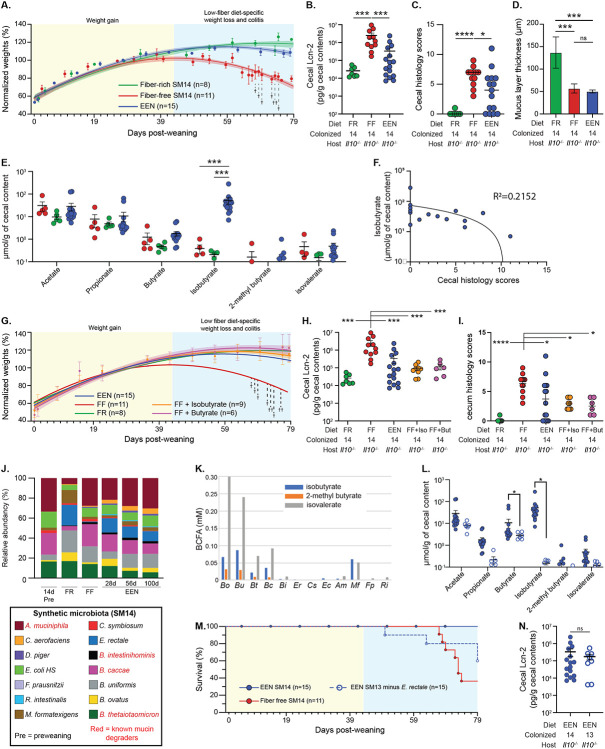
A fiber free exclusive enteral nutrition (EEN) diet improves inflammation in part through isobutyrate production. **A.** Weight trajectories of mice weaned onto FR, FF, and EEN diets. **B.** Cecal lipocalin levels of mice weaned onto FF, FR, and EEN diets. (n=7-15, two-way ANOVA and post hoc test with Original FDR method of Benjamini and Hochberg) **C.** Cecal histology scores of the same mice shown in B. (n=7-15, two-way ANOVA and post hoc test with Original FDR method of Benjamini and Hochberg) **D.** Mucus thickness measurements in mice shown in B. and C. revealing that the EEN diet does promote mucus thinning despite dampening disease in some individuals. (n=5, two-way ANOVA and post hoc test with Original FDR method of Benjamini and Hochberg) **E.** Short-chain and branched-chain fatty acid measuements in mice fed FF, FR, or EEN diets. (n=5-15, two-way ANOVA and post hoc test with Original FDR method of Benjamini and Hochberg) **F.** Correlation between isobutyrate production and Lcn2 levels. **G.** Weight trajectories of mice fed the FF diet and water supplemented with either 35mM isobutyrate (orange) or butyrate (purple). (n=6-15, two-way ANOVA and post hoc test with Original FDR method of Benjamini and Hochberg) **H.** Cecal Lcn2 levels in the mice from panel G. **I.** Cecal histology scores of the mice shown in G. (n=6-15, two-way ANOVA and post hoc test with Original FDR method of Benjamini and Hochberg) **J.** SM14 community composition in mice fed the FR, FF and EEN diets. Note the similarity of preweaned mice to adults fed FF and the 250-fold increase in E. rectale after feeding the EEN diet. **K.** Short and branched chain fatty acid measurements in culture supernatants of indvidual SM14 bacteria. **L.** Short and branched chain fatty acid measurements in EEN fed mice colonized with either the full SM14 (closed symbols) or an SM13 that lacks *E. rectale*. (n=6-15, one-sample Student's t-test and Wilcoxon test) **M.** Survival of SM14 and SM13 (minus *E. rectale*) colonized mice on the FF and EEN diets. **N.** cecal Lcn2 measurements of the mice shown in M. (n=6-15, one-sample Student's t-test and Wilcoxon test) Data are represented as mean ± SEM. *p < 0.05; **p < 0.01; ***p < 0.001; ****p < 0.0001.

A notable characteristic of the FF diet that we used is that its macronutrient composition (Supplemental Table 1) resembles some exclusive enteral nutrition (EEN) diets that are used clinically to treat some IBD presentations. EEN diets often contain low or no fiber [35] and have proven to be effective at inducing IBD remission in humans, although the precise mechanism(s) of action is still unknown [36]. Since dysfunction in IL-10 signaling is just one of several host pathways implicated in IBD, it is possible that this particular immune signaling axis is sensitive to lack of fiber, whereas others are not and those are the ones that benefit from EEN. To determine if an EEN diet that lacks fiber promotes inflammation in our gnotobiotic *Il10^−/−^* model, we weaned SM14-colonized pups onto a commercial EEN diet, which is normally taken as a liquid but in this case was freeze-dried, pelleted and sterilized by gamma irradiation (water was provided *ad libitum*). The average weight trajectories of 15 mice (3 separate experiments) supported the conclusion that the low fiber EEN diet promotes some disease development, although not as severe as the FF diet (Fig. 6A). Cecal Lcn2 measurements and histology further revealed a wide amount of variation in the disease present in individual animals, with some mice resembling healthy FR-fed mice, some resembling diseased FF-fed mice and some intermediate (Fig. 6B,C). Interestingly, the EEN diet increased the proportion of IgA-coated bacteria as soon as 21dpw and conserved the high-coated population compared to mice fed FF (Extended Data Fig. 8), suggesting immuno-regulatory properties distinct from both the FR and FF diets. Despite experiencing less inflammation, the EEN mice still exhibited reduced mucus thickness, which we expected given the lack of fiber in the formula used (Fig. 6D). Measurements of short- and branched chain fatty acids revealed that mice fed the EEN diet had elevated amounts of the branch-chained fatty acid (BCFA) isobutyrate (Fig. 6E). While isobutyrate varied between 7-281 μMol/g cecal contents, high levels were only weakly correlated with low inflammation (Fig. 6F). Isobutyrate is an isomer of the more widely studied, anti-inflammatory short-chain fatty acid butyrate, which did not increase, and isobutyrate is derived from L-valine fermentation by certain gut bacteria [37]. Interestingly, the other two BCFAs (2-methyl butyrate and isovalerate) did not increase despite being present at similar abundance in soy and milk protein [38], the two dietary proteins used in the EEN formulation (Fig. 6E). This suggests that the increase in isobutyrate may not be attributable to increased bulk dietary protein fermentation, which would be expected to increase all three BCFAs. Providing either isobutyrate or butyrate (both 35 mM) in the drinking water of pups weaned onto the disease-promoting FF diet decreased weight loss (Fig. 6G) as well as the amount of inflammation observed (Fig. 6H,I), revealing that either of these molecules can offset the diet- and microbiome-induced damage observed in this model.

Feeding the EEN diet promoted changes in the composition of the SM14, which most notably included a ~250-fold increase in the levels of *E. rectale* (Fig. 6J). While this Firmicute is known to produce butyrate, measurements of culture supernatants from 13 of the SM species in medium supplemented with L-valine plus other peptides did not reveal that *E. rectale* produces isobutyurate under the medium conditions tested. Rather, this metabolite was produced by all 4 of the *Bacteroides, plus M. formatexigens* (Fig. 6K). Nevertheless, to determine if the large increase in *E. rectale* abundance was functionally connected with production of isobutyrate, we bred neonatal mice born to parents harboring the SM14 community but lacking *E. rectale* (“SM13 minus *E. rectale*”) and fed them the EEN diet. Consistent with a causal role (indirect or direct) for *E. rectale* in isobutyrate production, mice lacking *E. rectale* failed to produce isobutyrate, as well as butyrate as expected (Fig. 6L). These mice also exhibited increased lethality (62.5% survival at 100d) relative to EEN fed mice harboring the full SM14 (Fig. 6M), although there was not a significant increase in cecal Lcn2 levels relative to the EEN/SM14 group (Fig. 6N).

## Summary and prospectus

The pathophysiology of IBD is complex and variable, in part because of the large number of different genetic contributions that combine with diverse environmental, microbial and dietary triggers that are known or hypothesized to influence its development. The diet-driven inflammation model that was developed and investigated in this study provides both a case study, in which potential contributing factors to IBD can be explored at mechanistic levels, as well as a more general experimental paradigm in which host genetic, immunological, microbiota and dietary factors can be systematically manipulated to examine their effects on disease progression. As examples of spontaneous, genetically driven intestinal inflammation models continue to be identified and developed [39-41], the ability to work with these murine models in gnotobiotic conditions and with defined diets holds the potential to uncover foundational principles about this complex disease.

The link between low dietary fiber and compromised thickness and/or permeability of the mucus layer has been emerging through several studies [12-14]. Here, we make a direct connection between the dietary fiber-gut microbiome axis in the context of IBD genetics, albeit in a way that depends on the specific bacteria present (SM14 vs. SPF). The discovery that the EEN diet tested exerts partial protection against inflammation was unexpected but may be unsurprising given the complexity of diet–microbiome–host interactions in the gut. The EEN diet clearly holds the potential to elicit mucus layer erosion, as well as inflammation, in this murine model, which we attribute to its lack of fiber along with other potential contributing factors. However, the finding that isobutyrate production may partially offset the development of inflammation, in a manner that is dependent on the presence of *E. rectale*, provides exciting opportunities to explore new microbiome pathways that are both influenced by diet and can exert beneficial effects. Identifying the critical bacteria in our SM14 and their corresponding pathways for isobutyrate production will be essential and could illuminate why ~15% of patients given EEN do not respond [42], perhaps because they are missing these bacteria/pathways.

Many questions remain regarding the development of the multifactorial diseases encompassed under the term IBD. While host genetic susceptibility is a mostly permanent trait that is difficult to repair, an exception being stem cell transplant in children lacking IL-10 signaling [43], the microbiome and especially diet are factors that could be manipulated to delay or reverse disease. Ever since the discovery that rearing *Il10^−/−^* mice under germfree conditions abrogates inflammation [17], there has been substantial interest in finding specific bacteria or bacterial combinations that elicit inflammation when added back to these animals. This search has yielded significant insight into the contributions of bacteria with known pathogenic qualities, such as *Helicobacter* spp., *Enterococcus faecalis* and adherent invasive *E. coli* [16]. However, roles for specific commensal bacteria have also been established using gnotobiotic mice [44], albeit without a clear understanding of the mechanism(s) involved. Based on the data presented here, we propose a model that is focused more on the influence of positive and negative bacterial metabolic pathways and not on specific taxa. The rationale for this view is that metabolic pathways like mucin degradation can be present in diverse bacteria and these organisms may fulfill similar roles in eroding mucus. In contrast, strains of the same species can vary in key metabolic capabilities, including mucus degradation, which is known to vary substantially among some strains of *Bacteroides* [45] and likely other groups. If one considers the effects of the microbiome on IBD development to be a cumulative series of positive and negative stimuli caused by the particular behaviors and metabolites exhibited by the microbes present, it should be possible to optimize beneficial processes (*e.g.*, butyrate, isobutyrate) while reducing detrimental events like mucus erosion. While some of these levers can be manipulated through diet, a promising path for intervention may eventually include adding or replacing specific bacterial taxa within a person’s individual microbiota with more optimal strains or perhaps even those that are engineered to increase beneficial metabolites.

## Materials and Methods

### Animal models, colonization diet and sample processing

Animal experiments at the University of Michigan followed protocols approved by the University of Michigan Institutional Animal Care and Use Committee (IACUC) . Germ-free B6.129P2-Il10tm1Cgn/JZtm mice (Institut für Versuchstierkunde und Zentrales Tierlaboratorium, Hannover, Germany) and wild-type C57BL/6J (University of Bern, Switzerland) were bred at the animal facility of the University of Luxembourg and underwent protocols approved by the Animal Experimentation Ethics Committee of the University of Luxembourg and the Ministre de l’Agriculture, de la Viticulture et du Development rural du Grand-Duché du Luxembourg (LUPA 2020/20). Germfree males and females were colonized at 7–10 weeks of age and none of these mice was involved in any previous experiments/treatments. Mice were either housed alone or in groups of appropriate gender, litter, and dietary experiments. Food and autoclaved distilled water were provided ad libitum. Mice were weighed and monitored at least weekly for diarrhea, prolapses, and general health state and this was increased to daily monitoring in animals that began experiencing weight loss. The *Muc2^−/−^* mice, crossed to C57BL/6J background, were obtained from Leonard Augenlicht (Albert Einstein University). For the generation of *Il10^−/−^ Muc2^−/−^* double knockout (DKO), the single KO mice were crossed to obtain double heterozygous mice until generation of *Il10^−/−^ Muc2^+/−^*. Full double KO mice developed spontaneous prolapse in germ-free condition during prolonged breeding periods, thus, the *Il10^−/−^ Muc2^+/−^* mice were used to generate the double KO mice used for all experiments. Genotyping was performed by extracting DNA from ear punches (Transnetyx). Mice were gavaged with the synthetic microbiota at 6–8 weeks and proceeded to diet change as previously described. The synthetic microbiota bacteria were grown in their respective medium (Desai, 2016) or a modified YCFA medium [46] prior to community assembly for gavages. The bacteria were cultivated under anaerobic atmosphere maintained with a gas mixture (85% N_2_, 10% H_2_, 5% CO_2_) to an optical density (absorbance 600nm) ranging from 0.5 to 1.0. The communities were assembled by mixing equal volumes of each specific bacterium and aliquoted into sealed screw cap tubes with its own headspace. Each mouse was gavaged with 0.2 mL of its specific community, depending on the experiment, with freshly prepared inocula for two-three consecutive days. A humane endpoint was used for mice that lost ≥20% of their starting weight and these mice were counted as lethalities in the weight loss and survival curves shown. Animals were euthanized using CO_2_ asphyxiation for 5 minutes followed by cervical dislocation. The gastrointestinal tracts were retrieved quickly, to prevent autolysis, and the sections separated. Cecal and colon contents of each animal were flash frozen in liquid nitrogen and kept at −80°C until further use.

The FR diet (Lab Diet 5013) and FF diet (Envigo-Teklad TD.130343) have been previously described [12]. The EEN diet employed was Nestle Nutren 1.5, which was lyophilized and sent to Envigo rodent diets to be formed into pellets, bagged and gamma irradiated prior to use. Apple (Vitacel AF401), oat (Vitacel HF600-30) and wheat (Vitacel WF200) fibers were from J. Rettenmaier (Schoolcraft, MI, USA) and added to the FF diet at 7.5% w/w with a corresponding decrease in the amount of glucose in the FF diet. Soluble, highly digestible starch was provided by Cargill (Gel Instant 12412).

### DNA extraction

DNA from fecal and cecal samples were isolated using a bead-beating phenol: chloroform extraction method followed by DNeasy Blood & Tissue Kit (QIAGEN, USA). In short, samples were weighed between 10-50mg and combined with acid-washed glass beads (212-300mm; Sigma-Aldrich, USA), 500uL Buffer A (200 mM NaCl, 200 mM Tris, 20 mM EDTA, 210 uL SDS (20% w/v, filter-sterelized), and 500 uL phenol:chloroform (Thermo Fisher Scientific, USA). The samples were disrupted using a Mini-Beadbeater-16 (Biospec Products, USA) for 3 minutes at room temperature and centrifuged (10,000 rpm, 4°C, 3 minutes). The aqueous phase was recovered and mixed with an equal volume of phenol:chloroform by gentle inversion and centrifuged (10,000 rpm, 4°C, 3 minutes). The remaining aqueous phase was recovered and mixed with 500 μL of chloroform, mixed by gentle inversion, and centrifuged (10,000 rpm, 4°C, 3 minutes). Recovered aqueous phase was mixed with 1 volume of isopropanol and 1/10 volume of 3M sodium acetate, and stored at −80°C for 20 minutes for DNA precipitation. Samples were centrifuged (15000 rpm, 4°C, 20 minutes), supernatant discarded, washed with 70% ethanol, air-dried, and resuspended in nuclease-free water. The sample DNA extracts were further purified with DNeasy Blood & Tissue kit, following manufacturer protocol (QIAGEN).

### RNA extraction, reverse transcription and qPCR

Freshly retrieved ileal, cecal and distal colon tissues were transferred into RNAlater^™^ (QIAGEN) and kept at 4°C up to a week. Then, RNAlater^™^ was removed and tissues were stored at −80°C until further use. Frozen tissues were transferred into 1 mL of TRIzol reagent (Invitrogen^™^), homogeneized with a 5 mm metal bead on a bead beater for 8 min at 30Hz and centrifuged for 3 min at 13000 rpm, 4°C. The supernatant was recovered, mixed thoroughly with 200 μl of chloroform and incubated at room temperature for 2-3 min before a centrifigation for 15 min at 13000 rpm, 4°C. The aqueous phase was recovered, mixed again with an equal amount of chloroform and centrifuged for 15 min at 13000 rpm, 4°C. The aqueous phase was recovered, mixed by inversion with 500 μl of isopropanol, incubated for 10 min at room temperature, and centrifuged for 10 min at 13000 rpm, 4°C. The pellet was washed with 1 ml Ethanol 70% and centrifuged for 5 min at 10000 rpm. The supernatant was discarded and the pellet dried for 5-10 min at 37°C, resuspended with 50 μl nuclease-free water and incubated for 15 min at 56°C. Finally, samples were treated with DNase following the Thermo Scientific DNase1, RNase-free Protocol, and RNA were purified with the RNeasy Mini kit (QIAGEN) according to manufacturer instructions. Final RNA concentrations were quantified by Nanodrop.

### Lipocalin 2 (Lcn2) measurements in cecal and fecal contents

Frozen cecal and fecal samples were used to quantify the presence of lipocalin-2 protein (LCN2) by enzyme linked immunosorbent assay (ELISA). Samples previously frozen (cecal, −80°C, and fecal, −20°C) were weighed in new tubes between 5-5 mg. Samples were kept over dry ice during the weighing. Samples were resuspended in 1 mL PBS (pH 7.4), vortexed for 30s, and kept at 4°C overnight to homogenize. Samples were then extensively vortexed to a homogeneous solution. To measure the LCN-2 levels, a DuoSet mouse lipocalin-2/NGAL ELISA kit (R&D Biosystems, USA) was employed using several dilutions of sample homogeneous solution. Quantification was done following manufacturer protocol.

### Soluble IgA measurements in cecal and fecal contents

To measure soluble IgA levels, Nunc^®^ MaxiSorp^™^ 384 well plates (Sigma-Aldrich) were coated overnight with 10 ng/well rabbit anti-mouse IgA (Novus Biologicals, Bio-Techne NB7506) in 20 μl/well of carbonate-bicarbonate buffer (Sigma, Ref.: C3041). After four washes with Washing Buffer (1% Tween-20, 154mM Sodium Chloride and 10mM Trisma-base), 75μl of Blocking Buffer (15 mM Trizma-Acetate, 136 mM Sodium Chloride, 2 mM Potassium Chloride and 1% (w/v) BSA (Bovine Serum Albumin)). After 2h at room temperature, wells were washed again. Sample homogeneous solution and standards (mouse IgA Isotype Control UNLB, Southern Biotech, Imtec Diagnostics, Ref: 0106-01) were diluted in Dilution Buffer (15 mM Trizma-Acetate, 136 mM Sodium Chloride, 2 mM Potassium Chloride, 0.1% (w/v) Tween-20, and 1% BSA) and incubated into the plate at 20 μl/well, room temperature for 90 min. After washing, 20 μl/well of a Phosphatase Alkaline-conjugated goat anti-mouse IgA (Southern Biotech, Imtec diagnostics, Ref: 1040-04), diluted 1/1000 in Dilution Buffer, was added and incubated at room temperature for 90 min. After a final wash, 40 μl/well of substrate (1 phosphate tablet (Sigma, ref S0642-200 TAB) dissolved in 10 mL Substrate Buffer (1 mM 2-Amino-2-methyle-1-propanole, 0.1 mM MgCl2.6H2O)) was added. The plate was incubated at 37°C for 60 min before the absorbance was measured at 405 nm using an ELISA plate reader (SpectraMax Plus 384 Microplate Reader from Molecular Devices; Software: SoftMax Pro 7 Software, Molecular Devices). The IgA concentration was determined for each sample using the formulated standard curve.

### Short- and branched-chain fatty acid quantification

Short-chain and branched-chain fatty acids (SCFAs) standards mixture was obtained from Sigma (CRM46975). 13C-short chain fatty acid stool mixture (Sigma, SBR00035-1mL) was used as the internal standard (IS). Analytical reagent-grade 3-nitrophenylhydrazine (3NPH)·HCl (Cat#N21804), EDAC·HCl (Cat#341006); HPLC grade pyridine (Cat#270407); LC–MS grade acetonitrile (Cat#34851), water (Cat#270733), and formic acid (Cat#5438040450) were also purchased from Sigma–Aldrich. The working standard solutions were created by performing serial dilution from the 10mM stock solution down to nM range using freshly prepared 50% (v/v) aqueous acetonitrile in water. The chemical derivatization protocol was modified from Han et al. [47]. Briefly, 20μL of the working standard solutions or samples was mixed with 40μL of 200mM 3NPH in 50% aqueous acetonitrile, 120mM EDAC-6% (v/v) pyridine solution in the same solvent and 4μL of the IS in a Verex glass vial. The mixture was reacted at 40°C for 30 min. After reaction, 96μL of 0.1% formic acid in 10% acetonitrile solution was added to the mixture to quench the reaction. 30μL of the reaction solution was then transferred to a new HPLC vial and 2-μL aliquot of each solution was injected into the LC- MS/MS instrument. Each modified SCFA was optimized in Agilent MS for detection through Agilent Optimizer 2.0. All optimized SCFAs information was combined, and a LC-MRM MS method was created. Retention time for each SCFA was determined from two transitions. Then the MRM MS method was transformed into a dynamic MRM MS or dMRM MS method with all the RTs and MS information for the final LC-MS/MS acquisition method.

LC-MS/MS analysis was performed on the Agilent Technologies Triple Quad 6470 LC/MS system consist of 1290 Infinity II LC Flexible Pump (Quaternary Pump), 1290 Infinity II Multisampler, 1290 Infinity II Multicolumn Thermostat with 6 port valve and 6470 triple quad mass spectrometer. Agilent Masshunter Workstation Software LC/MS Data Acquisition for 6400 Series Triple Quadrupole MS with Version B.08.02 is used for calibration, compound optimization and sample data acquisition.

A Waters Acquity UPLC BEH TSS C18 column (2.1 x 100mm, 1.7μm) column was used with mobile phase A) consisting of 0.1% formic acid in water; mobile phase (B) consisting of 0.1% formic acid in acetonitrile. Gradient program: mobile phase (B) was held at 15% for 1 min, increased to 55% in 19 min, then to 99% in 20 min and held for 2 min before going to initial condition and held for 4 min. The column was at 40 °C and 2 μl of sample was injected into the LC-MS with a flow rate of 0.3 ml/min. Calibration of the 6470 MS was achieved through Agilent ESI-Low Concentration Tuning Mix. Source parameters: Gas temp 300 °C, Gas flow 5 l/min, Nebulizer 45 psi, Sheath gas temp 250 °C, Sheath gas flow 11 l/min, Capillary −3500 V, Delta EMV −200 V. Dynamic MRM scan type is used with 0.07 min peak width. dMRM transitions and other parameters for each compound were list in a separate sheet. Delta retention time of plus and minus 1 min, fragmentor of 40 eV and cell accelerator of 5 eV were incorporated in the method. Data analysis was performed by Agilent Mass Hunter Quantitative Analysis for QQQ B.10.00 for integration. Results were exported to CVS file for further analysis.

### Lamina propria cell extraction and flow cytometry analysis

Cecal and colonic lamina propria cells were extracted using the Lamina Propria Dissociation Kit and gentleMACS Dissociators (Miltenyi Biotec, Germany) according to the manufacturer’s instruction. After digestion, cells were resuspended in PB buffer (PBS, pH 7.2, 0.5 % BSA) and counted. For analysis of T cells and NK cells with the expression of transcription factors, the FOXP3/Transcription Factor Staining Buffer kit (eBioscences – 00-5523-00) was used along with the following anti-mouse antibodies: BV605-conjugated anti-CD4 (Biolegend, RM4-5; 1/700), BV650-conjugated anti-B220 (BD Biosciences, RA3-6B2; 1/88), BV711-conjugated anti-CD3 (Biolegend, 17A2; 1/88), BV780-conjugated anti-CD45 (BD Biosciences, 30-F11; 1/88), FITC-conjugated anti-CD335/NKp46 (Biolegend, 29A1.4; 1/100), PE-Cy5-conjugated anti-CD8 (Biolegend, 53-6.7; 1/700), eF450-conjugated anti-FoxP3 (eBiosciences, FJK-16s; 1/200), PE-conjugated anti-GATA3 (Biolegend, 16E10A23; 1/44), PE-eF610-conjugated anti-EOMES (eBiosciences, Dan11mag; 1/100), PE-Cy7 -conjugated anti-Tbet (Biolegend, 4B10; 1/44), APC-conjugated anti-RORgt (eBiosciences, AFKJS-9; 1/22). For B cells analysis and immunoglobulin expression, the BD Cytofix/Cytoperm^™^ Fixation/Permeabilization Solution Kit (BD Biosciences – 554714) was used along with the following anti-mouse antibodies: eF450-conjugated anti-B220/CD45R (eBiosciences, RA3-6B2; 1/700), eF506-conjugated anti-CD19 (eBiosciences, 1D3; 1/88), BV711-conjugated anti-CD3 (Biolegend, 17A2; 1/88), BV780-conjugated anti-CD45 (BD Biosciences, 30-F11; 1/88), APC-conjugated anti-CD138 (Biolegend, 281-2; 1/100), FITC-conjugated anti-IgA (eBiosciences, mA-6E1; 1/700), PerCP-Cy5.5-conjugated anti-IgD (Biolegend, 11-26c.2a; 1/200), PE-conjugated anti-IgE (Biolegend, RME-1; 1/44), PE-Cy5-conjugated anti-IgM (BD Biosciences, R6-60.2; 1/100), PE-Cy7 -conjugated anti-IgG (Biolegend, Poly4053; 1/44). Breifly, 1.5 million cells were washed twice with PBS prior to 15 min of staining with the Zombie NIR^™^ Fixable Viability dye (BioLegend – 423105), followed by 2 washes with FACS Buffer (PBS, 5% fetal bovine serum) and fixation according to manufacturer instructions. Cells were then incubated at 4°C for 15 min with Fc Block (Rat anti-mouse CD16 and CD32; BD Pharmingen – Cat.553141) and 30 min with the antibody mixes. Both Fc Block and antibodies were diluted in the permeabilization buffer provided with the fixation kits. Finally, cells were washed twice in their respective permeabilization buffer and resuspended in FACS Buffer for acquisition on a NovoCyte Quanteon Flow Cytometer System (Agilent). The data were then analyzed on FlowJo.

### Analysis of IgA-coating of bacteria and sorting

Frozen fecal samples were homogeneized in 1 ml ice-cold PBS and centrifuged for 3 min at 100g, 4°C. Supernatant was filtered through a 70 μm straining sieve and centrifuged for 5 min at 10,000g, 4 °C. The pellet was resupended in 1 ml ice-cold PBS, the OD_600_ detected on a Nanodrop and the amount of bacteria computed as follow: 2 OD_600_ = 10^9^ bacteria. Bacteria were pelleted again for 5 min at 10,000g, 4 °C, and resuspended in 500 μl of staining buffer (PBS, 5% goat serum). After 20 min of incubation on ice, 1x10^9^ bacteria were washed with 1 ml ice-cold PBS and stained for 30 min at 4°C with 4 μg of FITC-conjugated anti-mouse IgA antibody (Southern Biotech) in 100 μl of staining buffer. Cells were then washed once and resupended in 1 ml PBS, and 100 μl of bacteria was pelleted and frozen until further analysis. Remaining bacteria where centrifuged and resuspended in 90 μl of staining buffer and 10 μL of anti-FITC MicroBeads. After 15 minutes of incubation at 4°C, bacteria were washed, resuspended in 500 μl of staining buffer and applied onto a LS column for sorting of IgA^+^ and IgA^−^ fractions with a QuadroMACS^™^ Separator (Miltenyi Biotec). IgA-coated and IgA-uncoated fractions were centrifuged and dry pellets were stored at −80°C until further analysis. For analysis of IgA coating of bacteria, frozen pellets were defrost on ice and washed with 1 ml of staining buffer. Since all samples were not sorted at the same time, bacteria were stained again for 30 min with 0.5 μg of FITC-conjugated anti-mouse IgA antibody (Southern Biotech) in 100 μl of staining buffer to refresh and harmonize the staining between batches. After a washing step, DNA was stained for 20 minutes with diluted 1:4000 in 200 μl of DNA staining solution (0.1 M HEPES, 0.9 % NaCl, pH 7.2). Finally, bacteria were washed twice with PBS, fixed for 20 min in 4% PFA, washed again and analyzed on a NovoCyte Quanteon Flow Cytometer System (Agilent). Data were then analyzed on FlowJo. For analysis of IgA affinity, bacteria were treated with 200 μl of PBS containing 3M NaSCN for 15 min at 4°C, 800rpm, prior to blocking and staining with FITC-conjugated anti-mouse IgA antibody as mentioned above.

### Mucus measurements

The colons were sectioned from the colon-cecum junction to the anus, and immediately fixed in freshly made Carnoy’s fixative (methanol:chloroform:glacial acetic acid, 60:30:10 v/v). The distal part of the small intestine was fixed in freshly made Carnoy’s fixative together with the half blunt end of the cecum. Fixed tissues were kept in Carnoy’s fixative for three hours and exchanged for fresh fixative for another 24 hours. The tissues were then washed in 100% methanol and kept until placed in cassettes for histology preparation. The remaining empty half of the cecum were flash frozen in liquid nitrogen and kept at −80°C until further use.

Slides were deparaffinized by submerging in xylene (Sigma-Aldrich, USA) for five minutes, followed by another xylene incubation for five minutes. Afterward, the slides were dehydrated twice in 100% ethanol for 5 minutes. The slides were then quickly washed in Milli-Q water and antigens were retrieved by submerging in antigen retrieving solution (10 mM sodium citrate, pH 6.0). The submerged sections were heated to 90°C for 10 minutes and cooldown in room temperature for 20 minutes. Slides were quickly dipped three times in Milli-Q water and blotted to remove excess liquid. To better hold liquid, a PAP pen was used to draw around the tissue area for the subsequent steps. The sections were blocked by covering the tissue in blocking buffer (1:10 goat serum (Sigma, USA) in Tris-buffered Saline (TBS; 500 mM NaCl, 50 mM Tris, pH 7.4)) and incubated for an hour at room temperature. For the primary antibody staining, the tissue was covered with a 1:200 dilution of Mucin 2 antibody (H-300) (Santa Cruz Biotechnology, USA) in blocking buffer and incubated for two hours at room temperature. Following the incubation, the slides were rinsed three times in TBS, for five minutes each. The secondary antibody staining was performed by covering the tissue with a 1:200 dilution of Alexa Fluor 488 conjugated goat anti-rabbit IgG antibody (Thermo Fisher Scientific, USA) in blocking buffer for one hour at room temperature in dark. The tissue sections were washed twice in TBS for 5 minutes, gently blotted, and covered with ProLong Gold Antifade reagent with DAPI (Invitrogen, USA), covered with cover slips and sealed with nail polish. The slides were kept at room temperature for 24 hours in dark, then kept in 4°C until imaging. The mucus layer in the sections were visualized using a Zeiss Apotome by taking pictures across fecal pellets and stitching the images together to compose a single image. Mucus layer measurements were performed by using BacSpace as described by Earle et al.

### Histological examination of intestinal tissue sections

Extent of histologic lesions was scored on a semi-quantitative basis by a trained pathologist (KE) in a blinded fashion, using a modification of the scoring system of Bugni *et al.* [48]. Inflammation, epithelial damage, epithelial hyperplasia and dysplasia and the presence or absence of submucosa edema were scored on a scale of 1-4 according to the following table. Scores for each category were added to determine a total score. Cecum and colon were scored separately.

**Table:** 

Score	0	1	2	3	4
**inflammation**	Normal	Scattered PMNs or plasma cells in lamina propria or submucosa	Coalescing mucosal and/or submucosal inflammation	Widespread submucosal inflammation	Severe diffuse transmural inflammation
**epithelial damage**	None	Focally dilated glands and/or attenuated surface epithelium, and/or clusters of sloughed cells in lumen	Focally extensive gland dilation and/or surface epithelial attenuation, many sloughed cells in lumen	Mucosa erosions	Mucosal ulceration
**hyperplasia/dysplasia**	Normal	Hypertrophy and/or hyperplasia present	Cellular and/or glandular dysplasia present		
**Submucosal edema**	Absent	Present			

### Mucus penetrability assay

Penetrability of the colonic mucus was assessed as described by Gustafsson et al [21]. Briefly, colons were flushed with ice-cold oxygenated KREBS Buffer (116 mM NaCl, 1.3 mM CaCl_2_, 3.6 mM KCl, 1.4 mM KH_2_PO_4_, 23 mM NaHCO_3_, and 1.2 MgSO_4_ – Carl Roth) and opened along the mesenteric axis. The longitudinal muscle layer was removed by blunt dissection and the distal mucosa was inserted in a perfusion chamber. The basolateral chamber was filled with 0.6 μg/ml SYTO9 (Fisher Scientific - 10237582) in oxygenated KREBS Glucose Buffer (KREBS Buffer containing 10mM Glucose, 5.7 mM sodium pyruvate and 5.1 mM sodium-l-glutamate), and the apical chamber was filled with oxygenated KREBS Mannitol Buffer (KREBS Buffer, containing 10mM Mannitol, 5.7 mM sodium pyruvate and 5.1 mM sodium-l-glutamate). After 10 min incubation in the dark at room temperature, FluoSphere^™^ carboxylate beads (1μm, red 580/605 – Invitrogen – F882) were applied on top and let to sediment on the tissue for 5 min in the dark at room temperature. The apical chamber was then gently washed several times to remove excess of beads. The chamber was incubated for 10 min in the dark before being visualized with a microscope. For each tissue, 4-7 confocal images were taken in XY stacks from the epithelium at the bottom to the beads on top, with 5 μm-intervals between sections. Images were then analyzed with Imaris software, and the penetrability was computed by comparing the distance between the outer border of the beads and the epithelium with the distance between the most inner beads and the epithelium.

### 16S rRNA gene-based community analysis

PCR and library preparation were performed by the University of Michigan Microbiome Core lab as described by Kozich et al. [49]. The V4 region of the 16S rRNA gene was amplified using the dual-index primers described by Kozich et al, 2013. The normalized and amplicon size evaluated samples were sequenced using an Illumina MiSeq. The raw sequences were analyzed using mothur v1.42.3 [50] with the included controls: PBS control during the sequencing and ZymoBIOMICS Microbial Community DNA Standard (cat# D6306) for error analysis. Sequences were aligned to the reference Silva database version 132 for contamination analysis and SPF experiments. Moreover, gnotobiotic mice sequences were also aligned to a custom reference database containing the 16S v4 region from each of the 14 bacteria members. The R package “vegan” was used to calculate the principal component analysis from the Bray-Curtis dissimilarity index.

## Extended Data

## Supplementary Material

1
