# Supplementary material for: Unravelling specific diet and gut microbial contributions to inflammatory bowel disease
Source: Res Sq. 2023 Mar 13:rs.3.rs-2518251. Preprint. [Version 1] doi: 10.21203/rs.3.rs-2518251/v1 (PMC10055531; doi:10.21203/rs.3.rs-2518251/v1)
Supplement: 1 [file NIHPPrs2518251v1-supplement-1.pdf]

Table S1. Nutritional and ingredient comparison between fiber free (FF) diet and Exclusive Enteral Nutrition diet Nestle Nutren 1.5

	Fiber free (FF) diet (% kcal from)	Nestle Nutren 1.5 (% kcal from)
**Protein**	23.6	18
**Fat**	34	35
**Carbohydrate (total)**	42.4	47
**Total:**	**100**	**100**
*Fiber %	8	0
Accessible fiber%	0	0
**Main ingredients:**	glucose (44.4%)	corn syrup (glucose/fructose)
(Descending order)	casein (26.9%)	maltodextrin
	fat from corn oil (7.5%), lard (7.5%)	canola oil
	cellulose (8%)	soy protein isolate
		sodium caseinate
		medium chain triglycerides (<2%)
		calcium caseinate
	(vitamin, minerals, antioxidants, and stabilizers not shown)	(vitamin, minerals, antioxidants, and stabilizers not shown)

*Note the only fiber added to the FF diet is crystalline cellulose as a bulking agent and is not known be accessible by any SM14 bacteria.
